# Molecular Dynamics Simulations of the Cardiac Troponin Complex Performed with FRET Distances as Restraints

**DOI:** 10.1371/journal.pone.0087135

**Published:** 2014-02-18

**Authors:** Jayant James Jayasundar, Jun Xing, John M. Robinson, Herbert C. Cheung, Wen-Ji Dong

**Affiliations:** 1 Voiland School of Chemical Engineering and Bioengineering and The Department of Integrated Physiology and Neuroscience, Washington State University, Pullman, Washington, United States of America; 2 Department of Chemistry and Biochemistry, South Dakota State University, Brookings, South Dakota, United States of America; 3 The Department of Biochemistry and Molecular Genetics, University of Alabama at Birmingham, Birmingham, Alabama, United States of America; University of Akron, United States of America

## Abstract

Cardiac troponin (cTn) is the Ca^2+^-sensitive molecular switch that controls cardiac muscle activation and relaxation. However, the molecular detail of the switching mechanism and how the Ca^2+^ signal received at cardiac troponin C (cTnC) is communicated to cardiac troponin I (cTnI) are still elusive. To unravel the structural details of troponin switching, we performed ensemble Förster resonance energy transfer (FRET) measurements and molecular dynamic (MD) simulations of the cardiac troponin core domain complex. The distance distributions of forty five inter-residue pairs were obtained under Ca^2+^-free and saturating Ca^2+^ conditions from time-resolved FRET measurements. These distances were incorporated as restraints during the MD simulations of the cardiac troponin core domain. Compared to the Ca^2+^-saturated structure, the absence of regulatory Ca^2+^ perturbed the cTnC N-domain hydrophobic pocket which assumed a closed conformation. This event partially unfolded the cTnI regulatory region/switch. The absence of Ca^2+^, induced flexibility to the D/E linker and the cTnI inhibitory region, and rotated the cTnC N-domain with respect to rest of the troponin core domain. In the presence of saturating Ca^2+^ the above said phenomenon were absent. We postulate that the secondary structure perturbations experienced by the cTnI regulatory region held within the cTnC N-domain hydrophobic pocket, coupled with the rotation of the cTnC N-domain would control the cTnI mobile domain interaction with actin. Concomitantly the rotation of the cTnC N-domain and perturbation of the D/E linker rigidity would control the cTnI inhibitory region interaction with actin to effect muscle relaxation.

## Introduction

Striated muscle contraction is triggered by Ca^2+^ and regulated by the thin filament (TF). The TF is a filamentous structure composed of the heterotrimeric troponin complex and tropomyosin bound to the double helical actin filament [1], [2]. Among the TF proteins, troponin plays a central role in the Ca^2+^-activated regulation of the muscle. The heterotrimeric troponin from the cardiac muscle is formed by three individual proteins: troponin C (cTnC), troponin I (cTnI) and cardiac troponin T (cTnT). cTnC is the Ca^2+^ binding protein and cTnI is the actin binding protein. At low levels of Ca^2+^, two regions of troponin I, the inhibitory region (cTnI-Ir) and the mobile domain (cTnI-Md), bind to actin to inhibit actomyosin ATPase and relax the cardiac muscle. The cTnT anchors the troponin complex on the actin filament and also interacts with tropomyosin. In the relaxed cardiac muscle, the cTnI−actin interactions act as a regulatory switch to prevent cross-bridge formation between actin and the myosin head through steric blocking of myosin-binding sites on actin via tropomyosin [3]–[5]. To activate the actomyosin ATPase and to generate force, Ca^2+^ binds to cTnC and induces an interaction between the regulatory (switch) region (cTnI-Rr) of cTnI and a hydrophobic patch located in the N-domain of cTnC, thus leading to an open conformation in the N-domain of cTnC [6], [7]. Based on the “drag and release” mechanism [8], this strong interaction pulls the inhibitory region of cTnI away from actin and relieves the inhibition of cTnI on actin. This paves the way for the movement of tropomyosin on actin surface. Tropomyosin moves from the blocked to the closed state thus exposing the myosin binding sites on actin for the myosin. Myosin binds to actin and generates force [9] which enable the heart muscle to pump blood. Though these known protein-protein interactions partially shed light on the Ca^2+^-based thin filament regulation, the molecular details of how the Ca^2+^ signal received at cTnC is transmitted to cTnI are still not well understood.

To fully understand Ca^2+^ regulation and cardiac muscle functioning, it is important to elucidate the Ca^2+^-induced changes to the structure and the structural dynamics of the TF proteins, particularly the troponin complex. The cardiac troponin core domain structure at saturating Ca^2+^ was solved [10], but till today no high resolution structure of the cardiac troponin complex in the Ca^2+^-free state is available. Although the crystal structure of the cardiac troponin in Ca^2+^-bound state provides some detailed structural information of cTnI-cTnC interaction, a number of functionally important regions involved in Ca^2+^-regulation mechanism were absent from the structure. For example, the N-terminal extension (residue 1–32) of cTnI, which is an important region for protein kinase A phosphorylation, and the portions of the N- and C-domains of cTnT, which tethers troponin to the thin filament, were absent in the crystal structure. Structures of the inhibitory region (residues 137–148) and the mobile domain (residue 192–210) of cTnI, were not defined. The lack of such information on the structure and dynamics of these critical regions and no knowledge of the structure of the cardiac troponin complex in the Ca^2+^-free state pose a barrier in our understanding of cardiac troponin function. Based on the core domain structure of the cardiac troponin complex at saturating Ca^2+^, the authors proposed an activation model [10], in which a significant conformational change is expected for the C-domain of cTnI (commencing from cTnI-Ir till the cTnI-Md) in response to Ca^2+^ binding and dissociation. However, no experiment have supported such large anharmonic fluctuation, especially that of the cTnI-Rr. In an effort to understand dynamics of cardiac troponin in Ca^2+^ activation process, Tobacman's group has used deuteration studies to unravel the dynamics of the cardiac troponin at varying biochemical states [11]. But no details as to which regions of the cardiac troponin complex are involved in anharmonic motion were revealed. Recently, molecular modeling techniques were used in studying the dynamics of the cardiac troponin and skeletal troponin [12]. In their study, they found that the cardiac troponin D/E linker to be more flexible when compared to the skeletal troponin in the Ca^2+^-saturated state. They made no attempt to unravel the structure of the troponin complex in the Ca^2+^-free state. There is no detailed structural model available where by the structures of the cardiac troponin complex in the Ca^2+^-free and Ca^2+^-saturated states have been compared. Till today there is no model that can shed light as to how the Ca^2+^ signal received at cTnC is propagated to cTnI. This knowledge is very necessary because the cTnI in concert with tropomyosin regulate myosin-actin interaction. There are two segments of cTnI that bind to actin, the troponin I inhibitory region and the troponin I mobile domain. The molecular details of the conformational rearrangement within cTnC that convey the Ca^2+^ signal to these regions of cTnI to bind or dissociate from actin is still an unknown.

In this study, we take a new approach by using Förster resonance energy transfer (FRET) technique to measure distances within the cTn complex and come up with a model of the how the cardiac troponin functions. As with every structure determination technique there are advantages and drawbacks. NMR is a very good technique for structure determination but is generally limited by the size of the protein. Particularly with the size of troponin complex, the structural power of NMR is significantly limited. Generally, it is easy for NMR to work with each individual troponin subunit, but difficult to work with the whole troponin complex. Furthermore, NMR requires high concentration of proteins to get sufficient signals, which makes it more difficult to work with the troponin complex because of its low solubility and high tendency to precipitate at high concentration. Though FRET has lower resolution than NMR, it can be easily applied to large sized protein complexes and work with proteins at low concentration that is close to physiological conditions. Using FRET we were able to acquire some important structural information about the functionally important regions of the troponin complex, such as the N-terminal extension of cardiac troponin I, the mobile domain of cardiac troponin I and the C-terminal of cardiac troponin T, which were missing in crystal structure of cardiac troponin core domain. But just a bunch of distances determined by FRET will not mean much unless they can be translated to structural information. Therefore the acquired FRET distance and half-width information were applied as distance restraints and bond-energies in this study while we performed molecular dynamics simulations of the cardiac troponin complex in explicit solvent. Time-resolved FRET measurements were performed to acquire a total of 45 FRET distances between cTnI and cTnT and between cTnC and cTnI in the Ca^2+^-saturated and Ca^2+^-free states. The recovered FRET mean distances and half-widths, together with 4 additional FRET distances from literature (two in the Ca^2+^-saturated state and two in the Ca^2+^-free state), were used as distance restraints and bond-energies while performing molecular dynamics (MD) simulations of the existing cardiac troponin complex [10]. This approach of using FRET in combination with MD simulations yielded structures of the cardiac troponin complex in the Ca^2+^-saturated and Ca^2+^-free states. This approach helped gain insight into the differences in structure and dynamics of the cardiac troponin complex in the Ca^2+^-saturated and the Ca^2+^-free states. The results of our studies helped us unravel the structural pathway by which the Ca^2+^ signal received at cTnC is transmitted to cTnI.

## Results

### FRET measurements of inter/intra-protein distance distributions within the troponin complex

In the crystal structure of the core domain cardiac troponin complex [10], the functionally important N-terminal extension and inhibitory region of cTnI were either missing or not resolved. To provide realistic structural restrains for MD simulations, time-resolved FRET measurements were performed to determine inter-protein distances from selected sites of cTnI residues 5, 15, 17, 27, 30, 40, 43, 129, 131, 145, 151, 160, 167 to, cTnT residues 240, 276 and 288, and cTnC residues 12, 35, 89 and 93. Among these cTnI residues, 145 is located in the crystallographically unresolved inhibitory region and residues 5, 15, 17, 27, and 30 are located within the missing N-terminal extension of cTnI. The other residues are all located at well-defined regions in the Ca^2+^-saturated troponin crystal structure. Intra-protein FRET distances of cTnI, from each of the residues 129 and 151 located at the two ends of the inhibitory region to each of the residues 5, 15, 30 and 43 on the N-terminal of cTnI were determined. These measurements were performed in the troponin complexes at both the Ca^2+^-free and Ca^2+^-saturated states. In total 45 pairs of inter-/intra-protein FRET distance distributions were recovered from these measurements (meaning that 45 distances were obtained in the Ca^2+^ free state and 45 in the Ca^2+^ saturated state). When restrained MD simulation of the cardiac troponin complex [10] was performed with the experimentally acquired FRET distances and half-widths, we anticipated that the structure of the troponin complex locked in the Ca^2+^-saturated state to be pushed to the Ca^2+^-free state. Also we performed free MD simulations expecting it to yield information on the dynamics of the cardiac troponin complex.

**Figure 1:**
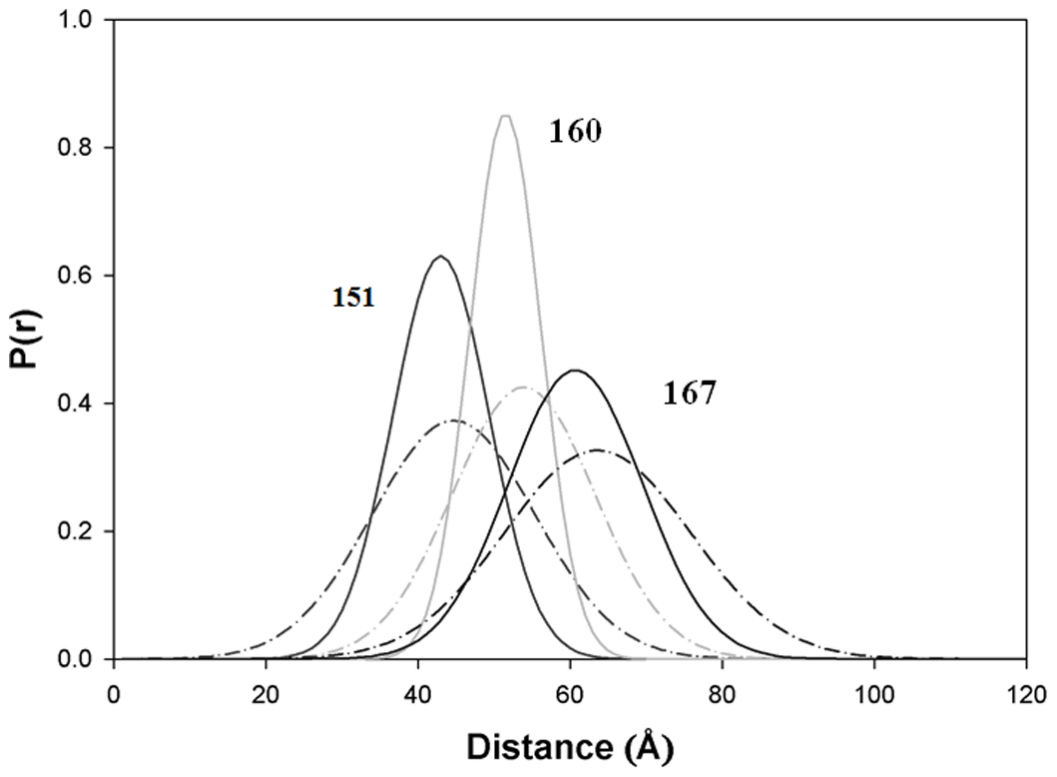
FRET distance distribution between cTnT and cTnI. Distribution of the distances P(r) between cTnT residue 276 and cTnI residues 151, 160, and 167, determined in the reconstituted cTn complex at low Ca^2+^ (broken curve) and saturating Ca^2+^ (solid curve).

In these FRET measurements, the distances between three cTnT residue (240, 276 and 288) located in the C-terminus of cTnT and selected cTnI residues were measured using either AEDANS (5-(iodoacetamidoethyl) aminonaphthelene-1-sulfonic acid) or MIANS (2-(4′-maleimidylanilino)naphthalene-6-sulfonic acid) attached to the cTnI residues as FRET donors. DABM (4-dimethylaminophenylazophenyl-4′-Maleimide) or DDPM (N-(4-dimethylamino-3,5-dinitrophenyl)maleimide) attached to the cTnT residues served as FRET acceptors. During our FRET measurements, we found the distances between cTnI residue 131 and cTnT residues 276 and 288 were much shorter than others. To measure the short distances, the MIANS-DDPM pair was used because the R_0_ (∼29 Å) of this donor- acceptor pair is compatible to the measured inter-protein distances in the range of 20–29 Å. For all other inter-protein distance measurements between cTnT and cTnI (Table 1), the AEDANS-DABM pair was used, because its R_0_ ∼40 Å. This donor-acceptor pair was appropriate for measuring inter-site distances from about 25 Å to 75 Å. In these distance measurements, the donor (AEDANS) was covalently attached to the single-cysteine residues of cTnI and the acceptor (DABM) to the single-cysteine residues in cTnT. For FRET measurements of the intra-protein distances between cTnI, the cTnI residues 129 and 150 were mutated to tryptophan, which served as the energy donor. The cTnI residues 5, 15, 30 and 43 of cTnI in the troponin complexes was mutated to cysteine and labeled with AEDANS which served as the FRET acceptor. To determine the distances between the N-terminal extension of cTnI and cTnC, we labeled each of the selected cTnI N-terminal residues with AEDANS, which served as the FRET donor, and labeled the residues of cTnC with the FRET acceptor, DDPM. Each selected donor and acceptor labeled proteins were reconstituted into a troponin complex and donor fluorescence intensity decays in the absence and presence of the acceptor were acquired at both Mg^2+^ (Ca^2+^-free) and at Ca^2+^-saturated states. The acquired fluorescence intensity decays were analyzed to derive FRET distance information in term of distance distributions [13]. A representative set of the distributions of the FRET distances between cTnT residue 276 and three cTnI residues 151, 160 and 167 is shown in Figure 1. These distributions, P(r), were recovered from the intensity decays of the donor using Eq. [2]. The distance at the peak of the distribution was taken as the mean distance between the donor and acceptor sites. For each distance, the distribution was slightly shifted toward shorter distance when the system was changed from the Ca^2+^-free state (relaxed) to the Ca^2+^-saturated state (activated). All recovered mean distances and half-widths are given in Table 1.

**Table 1:** FRET distance measurements within the cardiac troponin complex.

Acceptor	Donor	Ca^2+^-saturated state		Ca^2+^-free state	
TnT-240	TnI-	FRET(HW) Å	11.1 ns Å	FRET(HW) Å	9.5 ns Å
DABMI	131 AEDANS	48.1(10.2) *38.13*	50.89	47 (11.7)	48.49
DABMI	145 AEDANS	55.6(14.3)	54.17	58.1(14.4)	59.29
DABMI	151 AEDANS	66.9(12.8) *60.52*	67.34	63.9(18.0)	60.50
DABMI	160 AEDANS	73.3(18.1) *72.57*	73.37	70.7(21.8)	71.09
DABMI	167 AEDANS	78.4(19.9) *76.93*	79.49	76.7(35.5)	72.95
TnT-276	TnI-				
DDPM	131 MIANS	20.4(1.2) *18.21*	21.87	19.1 (3.6)	14.36
DABMI	145 AEDANS	34.3(11.2)	37.36	28.2(10.7)	32.79
DABMI	151 AEDANS	53.6(14.3) *45.24*	56.09	44.7(21.9)	41.60
DABMI	160 AEDANS	51.8(10.7) *57.12*	55.21	54.2(20.7)	52.56
DABMI	167 AEDANS	61.3(20.5) *66.12*	65.83	62.3(29.2)	60.43
DABMI	17 AEDANS	54.6(16.0)	53.26	50.0(15.4)	48.82
DABMI	27 AEDANS	45.1 (9.1)	44.92	47.4(11.0)	47.39
DABMI	40 AEDANS	38.0 (13.6)	40.00	39.4(19.3)	45.09
TnT-288	TnI-				
DDPM	131 MIANS	25.9(4.1)	21.87	26.1(8.8)	22.90
DABMI	145 AEDANS	39.5(17.8)	37.36	39.6(18.3)	40.39
DABMI	151 AEDANS	51.4(16.9)	56.09	50.1(22.2)	47.70
DABMI	160 AEDANS	55(21.3)	55.21	57.8(22.9)	58.65
DABMI	167 AEDANS	65.4(29.2)	65.83	63.2(26.3)	65.03
DABMI	5 AEDANS	54.9 (5.6)	58.59	56.8 (7.6)	57.81
DABMI	17 AEDANS	57.6(21.9)	55.99	55.6(20.1)	52.30
DABMI	40 AEDANS	46.9(14.2)	45.47	47.5(13.4)	47.06
TnC-12	TnI-				
DDPM	5 AEDANS	42.6(4.7)	42.07	45(7.5)	42.85
DDPM	15 AEDANS	39.9(3.3)	41.92	43.4(11.6)	44.38
DDPM	30 AEDANS	41.3(16.6)	41.24	42.1(16.5)	42.60
DDPM	43 AEDANS	38.8(16.3)	36.33	36.3(6.9)	35.65
TnC-35	TnI-				
DDPM	5 AEDANS	39.6(16.4)	35.54	33.7(2.8)	33.54
DDPM	15 AEDANS	24.9(11)	25.12	24.5(5.9)	24.77
DDPM	30 AEDANS	16(6.5)	14.76	19.2(7.3)	18.47
DDPM	43 AEDANS	35.5(4.1) *33.65*	31.10	36.2(4.4)	34.07
TnC-89	TnI-				
DDPM	5 AEDANS	34.1(15.5)	39.51	36.6(16)	40.73
DDPM	15 AEDANS	41.9(16.4)	39.52	34.5(2.3)	38.83
DDPM	30 AEDANS	45.2(16.8)	41.54	39.3(9.9)	38.19
DDPM	43 AEDANS	33.1(15.3) *21.43*	29.04	37.8(16.2)	37.0
TnC-93	TnI-				
DDPM	5 AEDANS	47.8(13.9)	40.08	38.7(16.3)	40.43
DDPM	15 AEDANS	33.8(15.4)	38.31	34.8(14.2)	37.44
DDPM	30 AEDANS	39.3(16.2)	40.42	39.8(16.5)	37.08
DDPM	43 AEDANS	28.9(3.6) *22.84*	24.99	39.1(14.4)	37.20
TnI-129	TnI-				
Trp	5 AEDANS	24.62(8.165)	33.85	41.2(20.4)	42.11
Trp	15 AEDANS	31.0(13.8)	27.75	32.1(2.5)	36.16
Trp	30 AEDANS	27.9(2.5)	27.33	30.4(12.1)	33.76
Trp	43 AEDANS	26.2(11.6) *33.22*	27.10	35.2(14.6)	40.39
TnI-150	TnI-				
Trp	5 AEDANS	31.2(1.2)	31.92	34.9(2.0)	34.76
Trp	15 AEDANS	31.8(1.2)	31.20	32.2(5.6)	31.51
Trp	30 AEDANS	29.7(10.5)	30.42	20.5(2.5)	25.77
Trp	43 AEDANS	26.9(3.1) *24.50*	27.42	29.1(10.9)	27.25
TnC-13C/51C [18]		31 *32.55*	31.20	25.8	25.49
TnI-5 [22] IAANS	TnI-192 Trp	51.5	52.06	46.8	46.94

The FRET distances measured between donor and acceptor probes in the Ca^2+^-saturated and the Ca^2+^-free states are tabulated. The half-widths are parenthesized. The FRET distances and restraints were applied as NOE restraints [49] between the Cα's of the participating amino acids. In columns 3,4 and 5,6 the experimentally measured FRET distance is tabulated along with the distance between the Cα's of the participating amino acids in the modeled structures, in the Ca^2+^-saturated states and Ca^2+^-free states respectively. In the FRET analysis, due to the ambiguity in the value of the dipole–dipole orientation factor between energy donor molecules and energy acceptor molecules and due to the dimensions of the probes attached by linkers to the side chains of the amino acid residues, the measured distance will have an uncertainty of ±10% [53]. Although the length of probe linkers is ∼10 Å, the linker is not unidirectional but folds randomly (during folding and rotation the probe and can acquire length of ∼7 Å). Based on these above factors there is good correlation between the measured FRET distance and the model. The italicized numbers in the third column pertain to the distance between the C-alphas in the crystal structure (1J1E). Compared to X-ray crystallography technique, FRET is a low resolution structural tool, and it does not have the lattice constraints that would be present in X-ray determined structure. However, FRET can acquire structural information in a more physiological environment, particularly with time-resolved approach (as we used here) it can provide dynamic information (represented by HW of the distance distribution) associated with each measured distance. Broad distributions of our FRET distances listed in Table 1 suggest that troponin exhibits a much dynamic structure in solution than in crystal. Therefore some discrepancies in the mean FRET distances with respect to the distances measured in X-ray structure would be expected. If we consider the structural dynamics (large HWs) observed in solution samples, these differences are in reasonable range.

### Modeling studies

**Figure 2:**
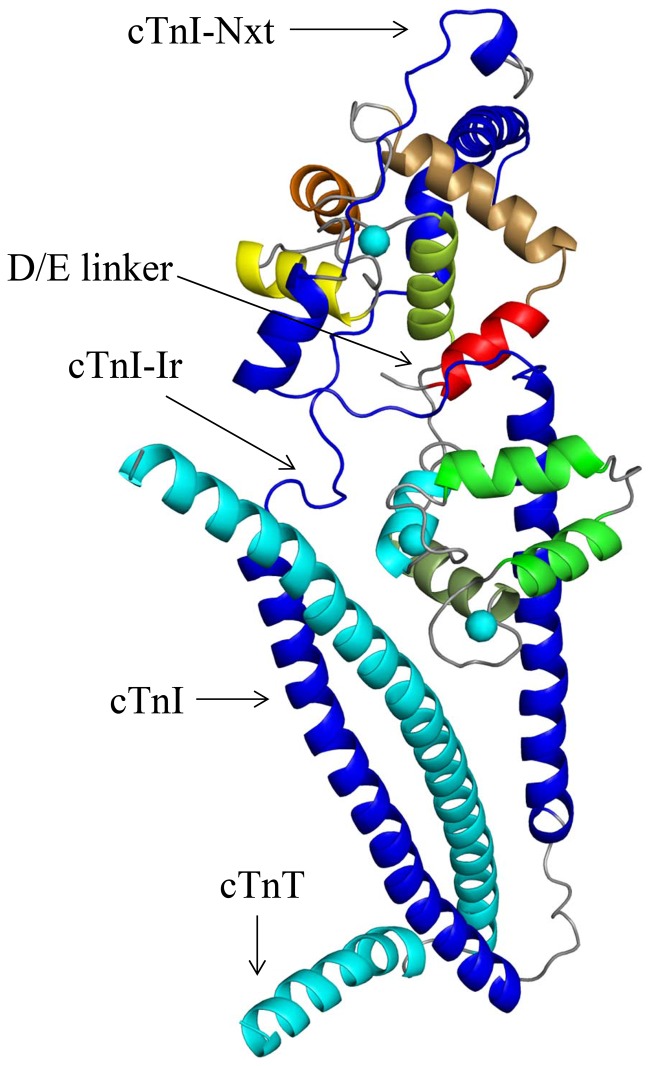
The starting structure for MD simulations. The crystal structure (1J1E.pdb [10]) of the cTn complex at saturating Ca^2+^ is depicted with the N-terminal extension of cTnI (cTnI-Nxt) docked over the cTnC N-domain. The N-terminal extension of cTnI was docked to the cTn complex using Hex, a protein-protein docking program. The cTnT and cTnI are colored cyan and blue respectively. The cTnC helices N, A, B, C, D in the N-domain are colored, red, mustard, orange, yellow and lime green. The bound Ca^2+^ ions at sites 2, 3, and 4 are rendered as spheres and colored cyan.

The distances and half-widths generated from the FRET experiments were used to model the cardiac troponin complex. The crystal structure of the cardiac troponin core domain that was resolved in the Ca^2+^-saturated state [10] did not have structural information pertaining to the physiologically important N-terminal and inhibitory regions of cTnI. To acquire this information and to shed light on the structure and structural dynamics of the cardiac troponin in the Ca^2+^-free and Ca^2+^-saturated states, we carried out MD simulations. The cardiac troponin core domain was simulated in three different conditions. The first was restrained MD simulations. During the restrained MD simulations the experimentally determined FRET distances and half-widths were applied as distance restraints and bond energies. This approach helped us select the starting structure shown in Fig. 2. The details of the validation process are detailed in the next paragraph. The second MD simulation was carried out in such a way that at the end of the first FRET distance restrained MD simulations, the restraints were removed and the simulation was allowed to continue for 250 ns. The results of this simulation give insight into the dynamics of the cTn complex. This helped us understand whether the cardiac troponin complex when in one biochemical state is always locked in that state, or if there are fluctuations in between states. The data is presented in figures S1, S2, S3. The third MD simulation of the cardiac troponin complex displayed in Fig. 2 was performed for 150 ns without any restraints. Since there was unfolding of cTnC helices in first simulation we wanted to show that the unfolding was due to the presence of restraints and was not because the force field was set up incorrectly. The results of the MD simulation of the troponin complex from the third set of simulations are given in the File S1. The data is presented in figures S4, S5, S6, S7.

There were two challenges in our modeling studies to overcome. The first was with regard to the location of the N-terminal extension of cTnI in the cTn complex. From previous NMR and peptide studies we concluded that the N-terminal extension of cTnI must be situated above the cTnC N-domain [14]–[17]. The second challenge was to decipher the biologically relevant conformation of the N-terminal extension of cTnI above the cTnC N-domain. To address these issues, based on the FRET and NMR experimental results from previous studies [14], [15], [17], [18] we formulated a set of criteria for validating the location and biologically relevant conformation of the N-terminal extension of cTnI in the cardiac troponin complex. The first criterion was, the N-terminal extension of cTnI must be located over the N-domain of cTnC. The second criterion was that only in the biologically relevant location and conformation of the N-terminal extension of cTnI over the cTnC N-domain, would the experimentally determined cTnC N-domain closing (in the Ca^2+^-free state) and opening (in Ca^2+^-saturated state) be observed [6]. In previous FRET experiments the closing and the opening of the cTnC N-domain, in low and saturating Ca^2+^ concentrations were detected by experimentally monitoring the distance between the cTnC residues 13 and 51 in the presence of cTnI [18]. Importantly, this phenomenon is observed in the crystal structure of the skeletal troponin complex too. Where, a 10 Å distance increase from 19.21 Å to 29.06 Å (measured between C-alphas), is observed between skeletal TnC residues 13 and 51 in the Ca^2+^-free (1YVO.pdb) and the Ca^2+^-saturated state structures (1YTZ.pdb). The distance increase was the result of Ca^2+^ binding to the skeletal troponin C which opened the hydrophobic pocket. Therefore, the above set of criteria used for validating the biologically relevant position of the N-terminal extension of cTnI in the cardiac troponin complex has sound experimental backing. After docking the NMR structure of the N-terminal extension of cTnI (PDB ID: 2JPW [16]) to the troponin complex, all the structures that complied with criterion 1 were retained and the rest discarded. The dephosphorylated N-terminal extension of cTnI was then ligated to the troponin complex via cTnI and the cardiac troponin complex was subjected to distance restrained molecular dynamic simulations. The structures that fulfilled criterion 1 were subjected to distance restrained energy minimization (EM), distance restrained simulated annealing (SA) and distance restrained MD simulations in the Ca^2+^-free state and Ca^2+^-saturated state. The response of the cTnC N-domain hydrophobic pocket to the presence of regulatory Ca^2+^ was monitored by measuring the distance between cTnC residues 13 and 51. We observed that in most of the structures that were subjected to the distance restrained simulations the cTnC N-domain hydrophobic pocket failed to open in the Ca^2+^-saturated state and failed to close in the Ca^2+^-free state, which are likely caused by incorrect docking of the N-terminal extension of cTnI over the cTnC N-domain. Therefore all structures that displayed this feature were discarded. After selection, the structure depicted in Fig. 2 was retained for further MD simulations.

### Restrained MD simulations of cardiac troponin in the Ca^2+^-free state

Simulation of the cardiac troponin complex (Figure 2) in the Ca^2+^-free state was carried out in the absence of regulatory Ca^2+^ at cTnC site 2. The Ca^2+^ in cTnC sites 3 and 4 were renamed as Mg^2+^ in the input coordinates file. Cardiac troponin C is a Ca^2+^ binding protein and has two domains, N- and C-domains, with three active Ca^2+^ binding sites. The site 2 on the N-domain binds specifically to only Ca^2+^ (the site 1 is an inactive site). The sites 3 and 4 on the C-domain bind to either Ca^2+^ or Mg^2+^. Under physiological conditions, it is likely that in the Ca^2+^ saturated state the C-domain is occupied by Ca^2+^, while in the Ca^2+^ free state, the C-domain binds Mg^2+^, but it may also binds to Ca^2+^ as suggested by literature [19]–[21] because of high Ca^2+^ binding affinity (10^−7^M). For clarity, we retained the Ca^2+^ in sites 2, 3 and 4 in the Ca^2+^ saturated state, and removed the Ca^2+^ from site 2 and renamed the Ca^2+^ to Mg^2+^ in sites 3 and 4 when modeling the Ca^2+^-free state.

**Figure 3:**
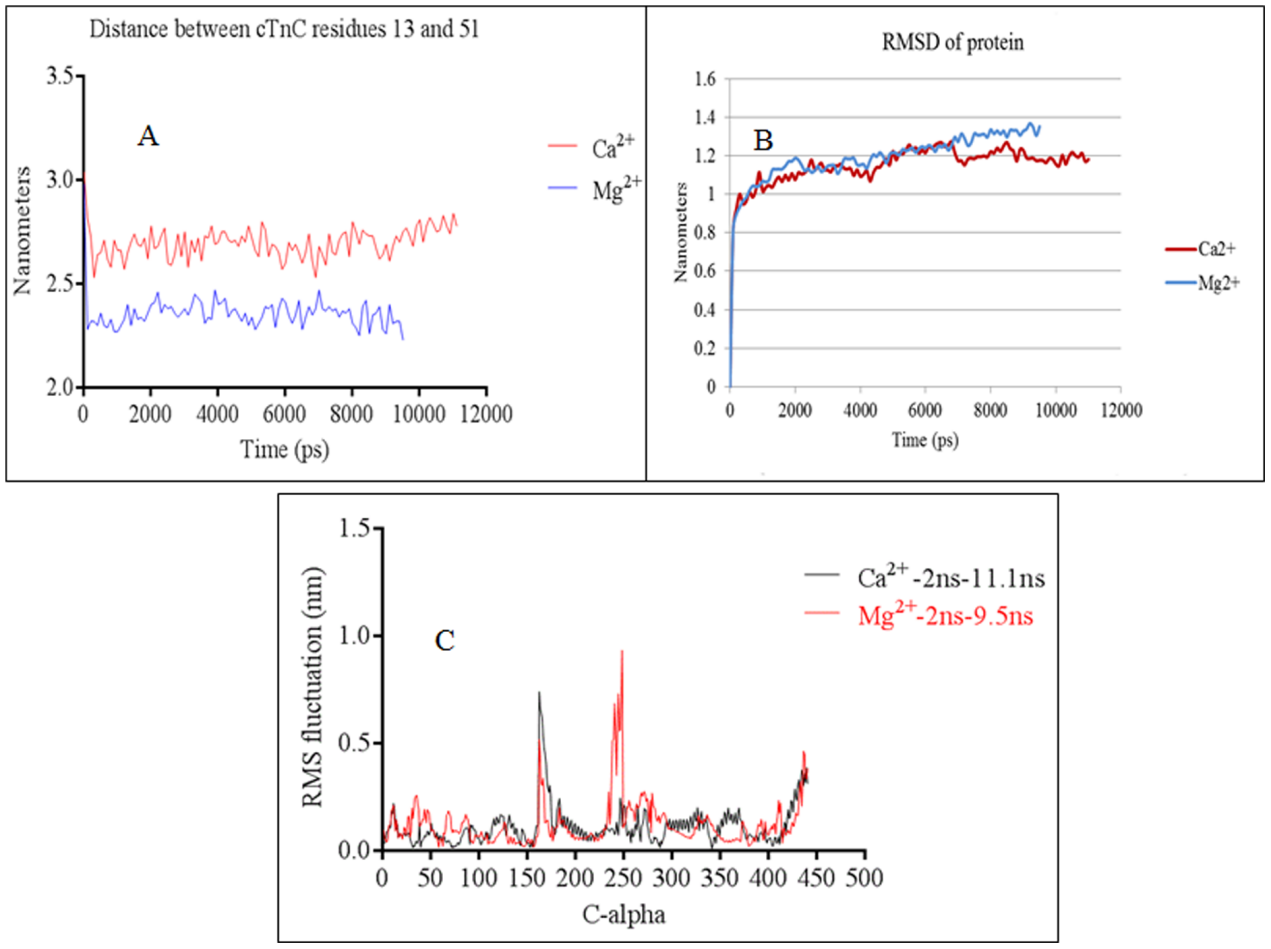
Data from MD simulations. (a) The opening and closing of the cTnC N-domain was monitored by measuring the distance between the Cα of cTnC residues 13 and 51. The residues 13 and 51 are located on helix A of cTnC and on the linker region of helices B and C, respectively. In the Ca^2+^-free state the distance between the two residues decreased because the cTnC N-domain hydrophobic pocket closed (due to the loss of Ca^2+^ from the cTnC site 2). In the Ca^2+^-saturated state, the distance between these two residues increased as they moved away from each other because the hydrophobic pocket opened (due to Ca^2+^ in cTnC site 2). The Y-axis represents the distance (in nanometers) between the cTnC residues 13 and 51. (b) Depicts that RMSD of the protein in the Ca^2+^-saturated and Ca^2+^-free states. (c) The root mean square fluctuations of the cardiac troponin complex was calculated after allowing the initial 2 ns for equilibration. In the graph the C-alphas from 1–161 pertain to cTnC, 162–249 pertain to cTnT, 250–442 pertain to cTnI. Fluctuations of more than 3 Å are observed between C-alphas 162–177 in both the Mg^2+^ (Ca^2+^ free) and Ca^2+^ saturated states. This pertains to cTnT N-terminal helix H1 (C-alpha 162–177 in the graph pertain to residues 202–217 in the crystal structure). Fluctuations are also observed towards the C-terminal end of cTnT helix H2 in the Mg^2+^ state (C-alpha 234–249 in the graph pertain to cTnT residues 273–288 in the crystal structure). Towards the end of the X-axis we can see that the C-terminal end of cTnI experiences fluctuations in both the biochemical states. This pertains to the cTnI-Md.

**Figure 4:**
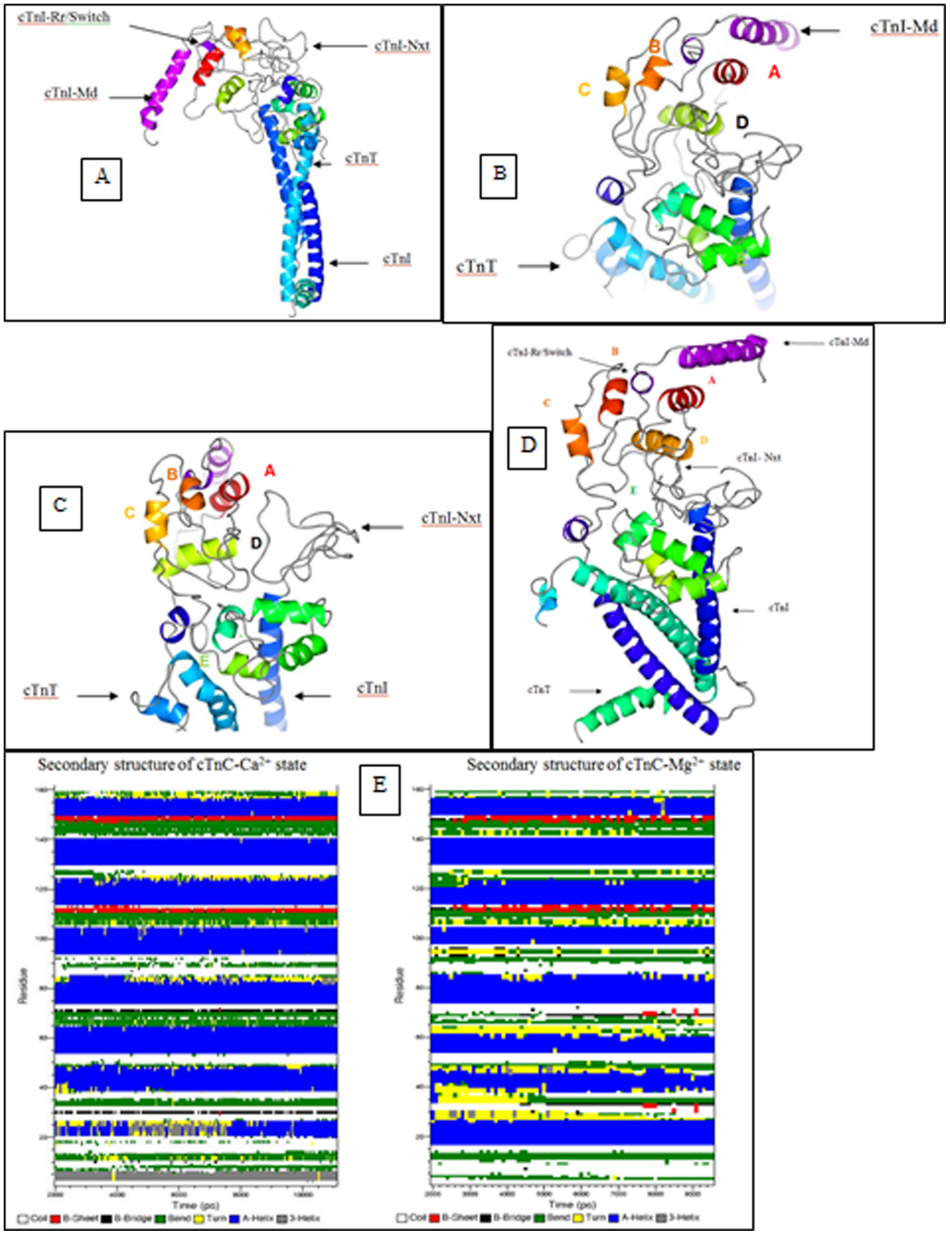
Ca^2+^-free state structure of the cardiac troponin complex. The cTn structure after 9.5^2+^-free state is depicted using CCP4MG version 2.7.3. (a) The cTn complex is positioned such as to see the dynamics of the cTnI-Md. The cTnI-Md is seen to have positioned itself close to the cTnC helix A. The cTnI-Rr held within the cTnC N-domain hydrophobic pocket has its secondary structure perturbed due to the closing of the hydrophobic pocket. (b) Depicts the cTnC N-domain wherein the loss of regulatory Ca^2+^ led to structural rearrangement of the helices B, C and D. The helices B and C are almost parallel to each other. (c) View of the N-terminal extension of cTnI above the cTn core domain complex. In this view the collapsed conformation of the cTnC N-domain hydrophobic pocket is well seen. It may be compared against the conformation of the open cTnC N-domain hydrophobic pocket in Fig. 5c. (d) The averaged structure of the cardiac troponin complex in the Ca^2+^ free state from 2 ns to 9.5 ns. The first two nanoseconds were given for the system to equilibrate. (e) The secondary structure of the troponin C in the presence of FRET distance restraints are calculated from 2 ns till 11.1 ns. The cTnC N-domain helices N (residues 4–11), A (residues 14–28), B (residues 38–47), C (residues 54–61) and D (residues 74–85) experienced considerable secondary structure evolution, but in contrast, the cTnC C-domain helices E (residues 94–104), F (residues 117–123), G (residues 130–140), and H (residues 150–157) are comparatively stable. Perturbation in the structure of helix D pulls and releases the D/E linker which in turn unfolds and refolds helix E. This fluctuations cause the D/E linker to alternate between flexible and rigid conformations that effectively helps release and retract the cTnI-Ir towards and away from actin in the absence and presence of Ca^2+^. The residue numbers associated with the helices of cTnC which are given within brackets were derived from the crystal structure 1J1E.pdb.

The experimentally measured FRET distance distributions in the Ca^2+^-free state (Table 1), were applied as distance restraints and bond energies while performing distance restrained EM, distance restrained SA and distance restrained MD simulations. In addition to the FRET distances listed in Table 1, FRET distances and half-widths from previous studies were also used as distance restraints and bond-energies in these distance-restrained simulations. They were the distances between cTnC residues 13 and 51 [6], [18] and the distance between cTnI residues 5 and 192 determined in the presence and absence of Ca^2+^ [22]. The Ca^2+^-free cardiac troponin complex was simulated for 9.5 ns under FRET distance restrains. The structure at the end of 9.5 ns is presented in Fig. 4. The simulated structure faithfully reproduced the closed cTnC N-domain hydrophobic pocket. The distance between residues 13 and 51 of cTnC decreased (Figure 3a) and this was consistent with previous experimental results [6], [18]. The root mean square deviation (RMSD) and root mean square fluctuations (RMSF) of the protein are presented in Figures 3b and 3c. Inspection of the simulated (Fig. 4a–c) and averaged structures (Fig. 4d) provided us detailed structural information on each of troponin subunit in the complex.

Upon comparing with the initial structure built from the Ca^2+^-saturated crystal structure of the troponin core domain (Figure 2), the cTnC N-domain helices in the Ca^2+^-free state experienced partial loss of secondary structure. For example, the helix N had totally lost its secondary structure and became fully unfolded and helix A was folded from Lys17-Ile26. The helices B and C were folded from Lys39-Val44 and Pro54-Met60, respectively, while helix D was folded from Phe74-Lys86. In the C-domain of cTnC, the helix E was folded from Ser98-Phe104. Interestingly, the helix E was unfolded between residues Glu94-Ser98. This made the D/E linker region less rigid when compared with the starting structure (Figure 2). The secondary structure of the remaining cTnC helices on the C-domain was conserved. The helices F and G were folded from Leu114-Thr124, and from Glu130-Gly140, respectively while helix H was folded from Tyr150-Phe156 (Figures 4a, 4b). Since the folding and unfolding of the helices of cTnC are critical in passing on the Ca^2+^ signal to cTnI, the time course evolution of the cTnC secondary structure is given in figure 4e.

In the cTnI subunit, the residues 1–40 that encompasses the N-terminal extension of cTnI adopted a random coil conformation. The first 30 amino acids of the N-terminal extension of cTnI were positioned in space above and in-between the two domain of cTnC. The interaction between cTnI and cTnC was observed to begin with the hydrogen bond between Glu32 of cTnC and Glu33 on the N-terminal extension of cTnI. Upon comparing with the starting Ca^2+^-saturated structure (Figure 2), the loss of regulatory Ca^2+^ led to the anharmonic fluctuation of the cTnI-Ir and the cTnI-Ir was positioned close to the cTnC helix C, and hydrogen bonds were observed between the two entities. At the downstream, the cTnI-Rr was partially unfolded, and the mobile domain located at the C-terminus of the cTnI-Rr moved down in space and was positioned towards cTnC helix A.

The cTnT subunit, predominantly adopted a helical conformation. The residues from Gln271 to Lys288 at the C-terminus end of the protein were partially unfolded and positioned away from the troponin complex (Figures 4c–d).

#### Hydrogen bonds in the Ca^2+^-free state

The hydrogen bonds found in this state may be classified into two categories, (i) Intra molecular hydrogen bonds and (ii) inter molecular hydrogen bonds. The list of these hydrogen bonds is given in Table 2. The hydrogen bonds were detected using Ligplot [23] and Rasmol [24]. As we analyzed the intra-molecular hydrogen bonds formed within cTnC, it was found that the loss of regulatory calcium not only caused the unfolding of helix N but also caused the N-domain of cTnC to rotate clockwise with respect to the C-domain. The rotation of the troponin C N-domain was observed in the skeletal troponin [25], [26]. Also in a recent study it was found that Ca^2+^ binding to cTnC causes the helix D making a counter clockwise movement [27]. So the binding of Ca^2+^ reverses the clockwise movement of the helix D. The clockwise rotation tilted the helix D towards the unfolded helix N. The tilting of the helix D caused bonds to be formed between Met1…Thr71, Asp2…Glu76. The unfolded helix N thrust Tyr5 towards the D/E linker. Also due to the partial unfolding of helix E there was a network of hydrogen bonds between the Tyr5(OH)…(NE)Arg83, Tyr5(OH)…(NZ)Lys94, and Tyr5(OH)… (OE2)Glu94. These bonds helped the helix N stay in close proximity to the D/E linker and the hydrogen bonds between Arg83 and Glu94 via Tyr5 helped keep the D/E linker from experiencing any major anharmonic fluctuation. The closing of the cTnC N-domain hydrophobic pocket led to hydrogen bond formations between cTnC residues Gly30…Val72, Asp33…Ser69, and Ser37…Ser69. These bonds were formed between the defunct site 1 and site 2 of cTnC. It is interesting to note that similar hydrogen bonds also exist in the crystal structure of cardiac troponin core domain complex (1J1E.pdb) between the above said sites (there are two hydrogen bonds between Val72 and Ile36 in the crystal structure and these hydrogen bonds helped keep the sites 1 and 2 close to each other). There was also a hydrogen bond between the Gly91 on the D/E linker and Glu161 of cTnC.

**Table 2:** Hydrogen bonds formed in the Ca^2+^-free state structure after 9.5 ns of simulations.

cTnC–cTnC	Comments
Met1(NH)…(O)Thr71	*H-bonds between the first amino acid of cTnC and the site 2 of cTnC.*
Asp2(NH)…(O)Glu76	H-bond between cTnC residue 2 and helix D.
Tyr5(O)…(HN)Arg83	H-bond between residue 5 and Arg82 situated at the base of helix D.
Tyr5(O)…(HN)Lys90	H-bond between residue 5 and lys90 situated at the middle of the DE linker.
Tyr5(O)…(O)Glu94	H-bond between residue 5 and Glu94 was possible due to the unfolding of helixE.
Gly30(O)…(HN)Val72	H-bond between sites 1 and 2.
Asp33(O)…(HN)Ser69	H-bond between sites 1 and 2.
Asp33(HN)…(O)Ser69	H-bond between sites 1 and 2.
Ser37(HN)…(O)Ser69	H-bond between sites 1 and 2.
Arg83(N)…(OE2)Glu94	H-bond formed between DE linker residues.
Gly91(NH)…(OE1)Glu161	H-bond between cTnC terminal residue and the DE linker.
Glu96(OE2)…(OH)Tyr150	H-bond between the unfolded helix E and helix H.
Glu161(OE1)…(HN)Gly91	H-bond between the cTnC C-terminal amino acid and the centre of the DE linker.
**cTnC—cTnI**	
Asp2(O)…(HN)Lys37	H-bond between cTnC residue 2 and the N-terminal extension of cTnI.
Asp3(O)…(HN)Lys51	H-bond between the N-terminal of cTnC and cTnI.
Glu32(O)…(NH)Glu33	H-bond between cTnC site2 residue and the N-terminal extension of cTnI.
Glu32(OE1)…(OG1)Thr32	H-bond between cTnC site2 residue and the N-terminal extension of cTnI.
Leu48(O)…(HN)Gln157	H-bond between cTnC and cTnI-Rr/switch.
Arg46(NH)…(O)Ala158	H-bond between cTnC and cTnI-Rr/switch.
Met60(O)…(HN)Arg146	H-bond between cTnC helix C and cTnI-Ir.
Asp62(NH)…(O)Thr144	H-bond between cTnC helix C and cTnI-Ir.
Asp65(NH)…(O)Lys139	H-bond between cTnC helix C and cTnI-Ir.
Asp67(OD1)…(ND1)His35	H-bond between cTnC site 2 and the N-terminal extension of cTnI.
Glu135(O)…(HN)Lys38	H-bond between cTnC helix G and the N-terminal extenion of cTnI.
Asp139(O)…(NH)Lys38	H-bond between cTnC helix G and the N-terminal extenion of cTnI.
Asp139(O)…(HN)Lys37	H-bond between cTnC helix G and the N-terminal extenion of cTnI.
Asn143(O)…(NE)Arg137	H-bond between cTnC site 4 and cTnI-Ir.
Asn143(OD1)…(NH)Gly138	H-bond between cTnC site 4 and cTnI-Ir.
Leu154(O)…(HN)Lys141	H-bond between cTnC helix H and the cTnI-Ir.
Phe156(O)…(HN)Lys37	H-bond between cTnC c-terminal and the N-terminal extension of cTnI.
**cTnC-cTnT-cTnI**	
TnC_Glu95(O)…(NH2)Arg267_TnT TnC_Glu95(OE2)…(N)Leu129_TnI	H-bond formed because of the unfolding of helix E. This region is seen as arching towards cTnI.
TnC_Glu96(OE2)…(NE)Arg267_TnT TnC_Glu96(OE1)…(NH2)Arg267_TnT TnC_Glu96(OE1)…(NH2)Ala127_TnI	H-bond formed because of the unfolding of helix E.
**cTnI-cTnT**	
Glu125(N)…(OG1)Thr284	H-bonds formed between the the unfolded region of cTnI 120–131.
Ile126(O)…(HN)Arg278	
Asp128(O)…(ND2)Asn271	
Gln131(OE1)…(N)Gln272	

The intra- and inter-molecular hydrogen bonds formed within the cTn complex in the Ca^2+^-free state after 9.5 ns of simulations.

Upon analyzing the hydrogen bonds formed between cTnC and cTnI we observed that the unfolding of helix N enabled hydrogen bond formation between cTnC Asp2 and cTnI Lys37 located on the N-terminal of cTnI and between cTnC Asp3 and Lys 51 of cTnI. Also Glu32 located on site 1 of cTnC hydrogen bonded with Glu33 and Thr32 on the N-terminal extension of cTnI. As we traverse from the N-terminus of cTnC towards the C-terminus, we observed hydrogen bonds between the loop, which connects helices B and C, with the cTnI-Rr. These hydrogen bonds held the cTnI-Rr close to the cTn complex and did not let the cTnI-Rr drop off the cTnC N-domain hydrophobic pocket (in short, there was no large spatial movement of the cTnI-Rr. This is in contrast to what is seen in the skeletal troponin (sTn) complex where the cTnI-Rr falls away from the skeletal troponin C (1YVO.pdb)). Due to the absence of regulatory Ca^2+^ the cTnI-Ir became more flexible and experienced anharmonic motion and moved close to the cTnC helix C. This resulted in hydrogen bonds between cTnC residues Met60, Asp62, Asp65 with cTnI residues Arg146, Thr144, Lys139 respectively. In the context of the thin filament the enhanced flexibility of the cTnI-Ir would serve to help it translocate towards actin rather than towards cTnC helix C. The cTnC residue Asp67 located on site 2 hydrogen bonded to cTnI His35 located on the N-terminal extension of cTnI. On the C-domain of cTnC, Glu135, Asp139 located on helix G, hydrogen bonded with cTnI residues Lys38 and lys37. Asn143 located on cTnC site 4, hydrogen bonded with cTnI residues Arg137, Gly138. Phe156 located on the cTnC H-helix hydrogen bonded with the Lys37 located on the N-terminal extension of cTnI.

In this state the cTnI residues Leu129-Asp135 formed a network of hydrogen bonds between cTnC-cTnI-cTnT. Looking at the network of hydrogen bonds in this region it led us to the conclusion that this is the region where the cTnI-Ir is tethered to the cTn complex. The hydrogen bond network may be seen in Table 2.

### Restrained MD simulations of cardiac troponin in the Ca^2+^saturated state

**Figure 5:**
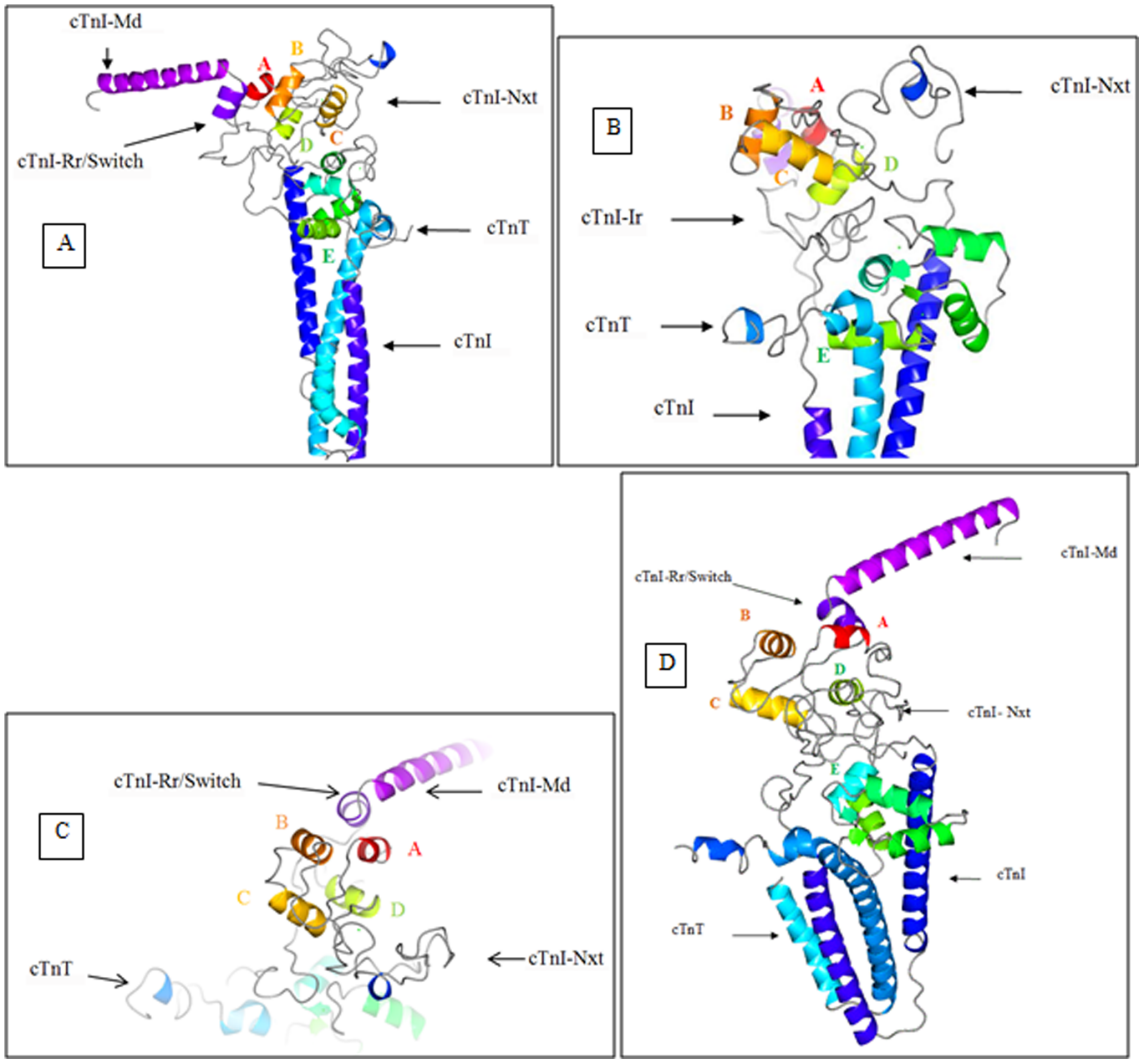
Ca^2+^-saturated state structure of the cardiac troponin complex. The cTn structure after 11.1^2+^-saturated state is depicted using CCP4MG version 2.7.3. (a) The secondary structure of the cTnI-Md and cTnI-Rr are conserved. The cTnI-Md points away from the cTn domain complex (b) The conformation of the helices B and C are almost orthogonal to each other. The helix D may be observed to have partially unfolded in this state. The structure of the N-terminal extension of cTnI above the cTn core domain complex is well seen. The N-extension of cTnI is seen interacting with sites 1 and 2 of cTnC. The C-terminus of cTnT is unfolded, probably an indication of its interaction with tropomyosin. The bound Ca^2+^ ions are seen as green dots. (c) Image of the cTnC N-domain as viewed from above the cTn complex. (d) The averaged structure of the cardiac troponin complex in the Ca^2+^ free state from 2 ns to 11.1 ns. The first two nanoseconds were given for the system to equilibrate.

The Ca^2+^-saturated state simulation of the cardiac troponin complex depicted in Figure 2 was performed by retaining all the bound Ca^2+^ ions from the crystal structure. The Ca^2+^-saturated FRET distances and half-widths from Table 1, as well as the two FRET distances from previous measurements [6], [18], [22], were applied as distance restraints and bond energies. The starting structure (Figure 2) was simulated for 11.1 ns with restraints. The structure at the end of 11.1 ns is presented in Figure 5.

The cTnC was the first entity that was analyzed. The helix N retained its helical conformation (in the Ca^2+^-free state the helix N was totally unfolded). The helix A was folded from Lys21-Ile26 (in the Ca^2+^-free state the helix A was folded from Lys17-Ile26), the helix B was folded from Glu40-Leu48 (in the Ca^2+^-free state the helix B was folded from Lys39-Val44), the helix C was folded from Pro54-Val64 (in the Ca^2+^-free state the helix C was folded from Pro54-Met60), the helix D was folded from Phe74-Met81 (in the Ca^2+^-free state it was folded from Phe74-Lys86), the helix E was folded from Glu94-Phe104 (in the Ca^2+^-free state it was folded between Ser98-Phe104), the helix F was folded from Leu114-Thr124, the helix G was folded from Glu130-Asp139 and the helix H was folded from Tyr150-Met157. During the simulations, the N-domain of cTnC maintained an open conformation. This was monitored by measuring the distance between cTnC residues 13 and 51 (Figure 3a). This feature was consistent with previous biochemical result [18]. The RMSD of the system in presented in Figure 3b. The time course evolution of the cTnC secondary structure is given in figure 4e and the analysis of root mean square fluctuations of the cardiac troponin complex in the Ca^2+^-saturated state is found in Figure 3c. The averaged structure of the cTn complex in the Ca^2+^ saturated state is presented in Figure 5d.

The second entity of the cTn complex is the cTnI. The N-terminal extension of cTnI adopted a random coil structure and was positioned partly above the N-domain of cTnC and in between the two domains of the cTn complex. Ala31 is closest to the N-domain of cTnC and interacted with the regulatory N-domain of cTnC by hydrogen bonding to Asp33. There were many hydrogen bonds that were observed between cTnI and cTnC, they are summarized in Table 2.

The third entity in the cTn complex is cTnT. This molecule predominantly maintained a helical secondary structure. This was however perturbed from residues Val262-Lys288 (in the Ca^2+^-free state the cTnT was unfolded from Gln271-Lys288) and the C-terminal end of cTnT was extended and positioned away from the cTn complex (Fig. 5b). The hydrogen bonds formed between cTnT and the other entities of the cardiac troponin complex are tabulated in Table 3.

**Table 3:** Hydrogen bonds formed in the Ca^2+^-saturated state structure after 11.1 ns of simulations.

cTnC–cTnC	Comments
Asp2(O)…(HN)…Arg83	H-bond between helix N and helix D.
Lys17(NZ)…(O)Met85	H-bond between helix A and DE linker.
Ala31(O)…(HN)Gln58	H-bond between site1 and helix C.
Ala31(NH)…(O)Gly70	H-bond between site 1 and 2.
Leu29(O)…(HN)Val72	H-bond between site 1 and 2.
**cTnC—cTnI**	
Gln16(O)…(HN)Ala152	*H-bond between the unfolded cTnC helix A and the N-terminus of cTnI-Rr/switch.*
Asp33(NH)…(O)Ala31	H-bond between cTnC siteI and cTnI N-terminal extension.
Asp62(OD1)…(ND1)His35	H-bond between cTnC helix C and N-terminal extension of cTnI.
Glu63(O)…(NZ)Lys139	H-bond between cTnC helix C and cTnI-Ir.
Asp65(O)…(NZ)Lys139	H-bond between cTnC helix C and cTnI-Ir.
Glu66(O)…(NZ)Lys139	H-bond between cTnC helix C and cTnI-Ir.
Asp67(O)…(HN)Ala36	H-bond between cTnC helix C and the N-terminal extension of cTnI.
Asp67(O)…(NH)His35	H-bond between cTnC helix C and the N-terminal extension of cTnI.
Gly68(O)…(NH)His35	H-bond between cTnC helix C and the N-terminal extension of cTnI.
Ser69(O)…(HN)Thr32	H-bond between cTnC helix C and the N-terminal extension of cTnI.
Ser84(O)…(O)Arg147	H-bond between cTnC base of cTnC helix D and cTnI-Ir.
Asp87(O)…(NH)Arg146	H-bond between the base of cTnC helix D and cTnI-Ir.
Lys138(O)…(HN)Lys37	H-bond between cTnC helix G and the N-terminal extension of cTnI.
Asp141(O)…(HN)Lys37	H-bond between cTnC helix G and the N-terminal extension of cTnI.
Asn144(O)…(HN)Lys37	H-bond between cTnC site 4 and the N-terminal extension of cTnI.
Asn143(O)…(HN)Leu36	H-bond between cTnC site 4 and the start of cTnI-Ir.
**cTnT-cTnC**	
Arg278(NH)…(OD2)Asp151	
Arg278(NE)…(OD1)Asp151	
**cTnT-cTnI**	
Gln273(NE2)…(O)Glu125	H-bonds formed between cTnT and cTnI site just N-terminus to the cTnI-Ir. It probably helps keep the cTnI pinned to the cTn complex.
Gln273(NE2)…(O)Ile126	

The intra- and inter-molecular hydrogen bonds formed within the cTn complex in the Ca^2+^-saturated state after 11.1 ns of simulations.

### Hydrogen bonds in the Ca^2+^-saturated state

After 11.1 ns of simulations we analyzed the hydrogen bonds formed within cTnC. Asp2 and Lys17 of cTnC had hydrogen bonded to cTnC Arg83 and Met85 located on helix D. cTnC residue Ala31 on the defunct cTnC site 1 hydrogen bonded to cTnC Gln58 on helix C. Also Ala31 and Leu29 on site 1 hydrogen bonded with Gly70 and Val72 on site 2 of cTnC, this helped maintain the sites 1 and 2 close to each other.

Next we analyzed the hydrogen bonds between cTnC and cTnI. The partial unfolding of helix A enabled Gln16 located on helix A to hydrogen bond with cTnI Ala 152 located at the beginning of the cTnI-Rr. Asp33 located on site 1 of cTnC hydrogen bonded with Ala31 of cTnI. Asp62 on helix C and Asp 67, Gly68, Ser 69 on cTnC site 2, hydrogen bonded with His35, Ala36, His35, and Thr32 located on the N-terminal extension of cTnI. We also observed that the residues Glu63, located on helix C and Asp65, Glu66 located towards cTnC site 2, hydrogen bonded to Lys139 located on the cTnI-Ir. As we traverse towards the C-terminus of cTnC, we observed Ser84 and Asp87 hydrogen bonding with Arg147 and Arg146 of cTnI. Towards the C-domain of cTnC we observed Lys138 located on helix G and Asp141, Asn143, Asn144 located on site 4, hydrogen bond with cTnI residues Lys37 and Leu36.

### Unrestrained MD simulation

Apart from the distance restrained simulations, two sets of unrestrained MD simulations were performed in the Ca^2+^-saturated and Ca^2+^-free states.

1. The troponin complex at the end of 9.5 ns (Ca^2+^-free state) and 11.1 ns (Ca^2+^-saturated state) were further simulated for 250 ns. This simulation was performed to understand the dynamics of the cardiac troponin complex in either biochemical states. These simulations are notated as simulations II.
2. The troponin complex was simulated without any restraints in the Ca^2+^-saturated state and Ca^2+^-free state. This simulation was conducted to show that the unfolding of helices in cTnC N-domain was due to the presence of restraints and not due to a flaw in us setting up the simulations. These simulations are notated as simulations III

Simulation II was conducted to see if there was any structural drifting in between the biochemical state with respect to the state that they were currently in. The distance restrained simulations gave us the structure of the cardiac troponin structure is in the Ca^2+^-free state and Ca^2+^-saturated state. Therefore by conducting free MD simulations it would give us an idea of what the dynamics of the troponin population in each biochemical condition would be. It was very clearly observed that in free simulations, the troponin complex does not stay in the Ca^2+^-free or Ca^2+^-saturated state for long. There is structural drifting between the biochemical states. It is known from the distance restrained simulations that in the presence of bound regulatory Ca^2+^ the cTnC N-domain must remain open. In the free simulations we observed that the despite the presence of regulatory Ca^2+^ bound in the N-domain of cTnC, the cTnC N-domain closes (File S1). In the absence of bound regulatory Ca^2+^ it is known that the cTnC N-domain should collapse. We find that cTnC N-domain does not always remain collapsed. But instead after a period of time the cTnC N-domain starts to open. This indicates that there is a varied population. In the saturating Ca^2+^ condition there is a population of cTn complex that sample the closed state and vice versa. This is supported by previous studies [27].

## Discussion

Knowing the detailed structure, kinetics and the dynamics of the TF proteins in response to Ca^2+^ binding and dissociating is extremely important, as this would help in designing drugs to tackle cardiomyopathies associated with the TF. To partially achieve this goal, in this study, we carried out restrained MD simulations of the cardiac troponin core domain. First, we determined the FRET distances and half-widths between those regions that were not resolved in the existing Ca^2+^-saturated state crystal structure to, the cardiac troponin core domain complex [10]. These distances were measured in the Ca^2+^-free and Ca^2+^-saturating conditions. Later, the experimentally acquired FRET distances and half-widths were applied as distance restraints and bond energies while simulating the cardiac troponin complex at both the Ca^2+^-free and Ca^2+^-saturated states. The restrained simulations were performed for 9.5 ns in the Ca^2+^-free state and 11.1 ns in the Ca^2+^-saturated state. Previously there was a modeling study where the troponin and tropomyosin was modeled in the Ca^2+^-free and Ca^2+^ saturated state, without actin [28]. Our modeling approach differs from theirs, as we had experimentally derived distance restraints. The distance restraints helped us position the cTnI-N-terminal extension above the N-domain of cTnC and this enabled the simulations to recreate the closing and opening of the cTnC-N-domain hydrophobic pocket in the absence and presence of Ca^2+^.

MD simulations of biological macromolecules are typically performed for a long period of time because the subject protein molecule needs to sample through all possible conformations. But there is no guarantee that the molecule has reached its global minima, especially when just free MD simulations are being used for structure refinement. Apart from the long time scales that the target protein is typically subjected to, the success of a simulation is also judged by whether the simulation reproduces the structural or dynamic features that have observed from experimental studies. In our studies we have circumvented long simulation time scales by applying experimentally determined restraints while performing MD simulations, because these restraints help guide the molecule in conformational space towards its biologically relevant conformation. Also the model does reproduce experimental features determined by experimental studies. This approach enabled us to build the final models of the cardiac troponin core domain complex in the Ca^2+^-free and Ca^2+^-saturated states.

The final models got from the distance restrained simulations provide novel insight into the structural changes that takes place in the cardiac troponin core domain in both of the Ca^2+^-free and Ca^2+^-saturated states. It also helped us understand how the Ca^2+^ signal is transmitted within the cardiac troponin complex.

### Structure of cardiac troponin C

Upon comparing the final distance restrained simulated structure of the Ca^2+^-free state to the structure of the Ca^2+^-saturated state, we observed a significant conformational rearrangement of the helices B and C of cTnC in the complex, which is accompanied by the conformational change of Ca^2+^ binding loop 2. In the Ca^2+^-saturated state Asp 67 and Glu76, the two terminal residues of cTnC Ca^2+^ binding loop 2, had a separation distance of 9.08 Å(measured between C-alphas). In the crystal structure (1J1E.pdb) the separation distance was 8.95 Å, whereas in the Ca^2+^-free state the separation distance increased to15.70 Å. This loss of Ca^2+^-induced structural change in the binding loop caused conformational rearrangement of helices B and C. These helices were nearly orthogonal to each other in the Ca^2+^-saturated state, whereas in the Ca^2+^-free state they were parallel. This conformational change has direct implication in the interaction between the cTnC hydrophobic patch and the regulatory region of cTnI because the rearrangement of helices B and C control the orientation of the cTnI-Rr held within the cTnC hydrophobic pocket (discussed under the *structure of cardiac troponin I*).

Another unique feature is the structural dynamics of the helices D and E of cTnC in response to Ca^2+^. The helix D in the Ca^2+^-free state assumed a helical conformation between residues 74–86, whereas in the Ca^2+^-saturated state the helix was confined to residue 74–81. In the Ca^2+^-saturated state the helix E was fully folded whereas in the Ca^2+^-free state the cTnC residues 94–97 of the helix E were unfolded. The Ca^2+^ based folding and unfolding of helix D is observed in the crystal structures of the cTnC N-domain in the Ca^2+^-free (1SPY.pdb [29]) and Ca^2+^-saturated states (1MXL.pdb [7]). Comparing these structures reveled that in the Ca^2+^-free state the helix D has a helical conformation from residues 73–83, whereas in the Ca^2+^ free state the helix D has a helical conformation from residues 73–85. The crystal structures certainly do reveal that the helix D tends to have a longer helix in the Ca^2+^-free state with respect to the Ca^2+^-saturated state.

This indicates that the Ca^2+^ based folding and unfolding of helix D observed in the crystal structure is being replicated in our model too.

**Figure 6:**
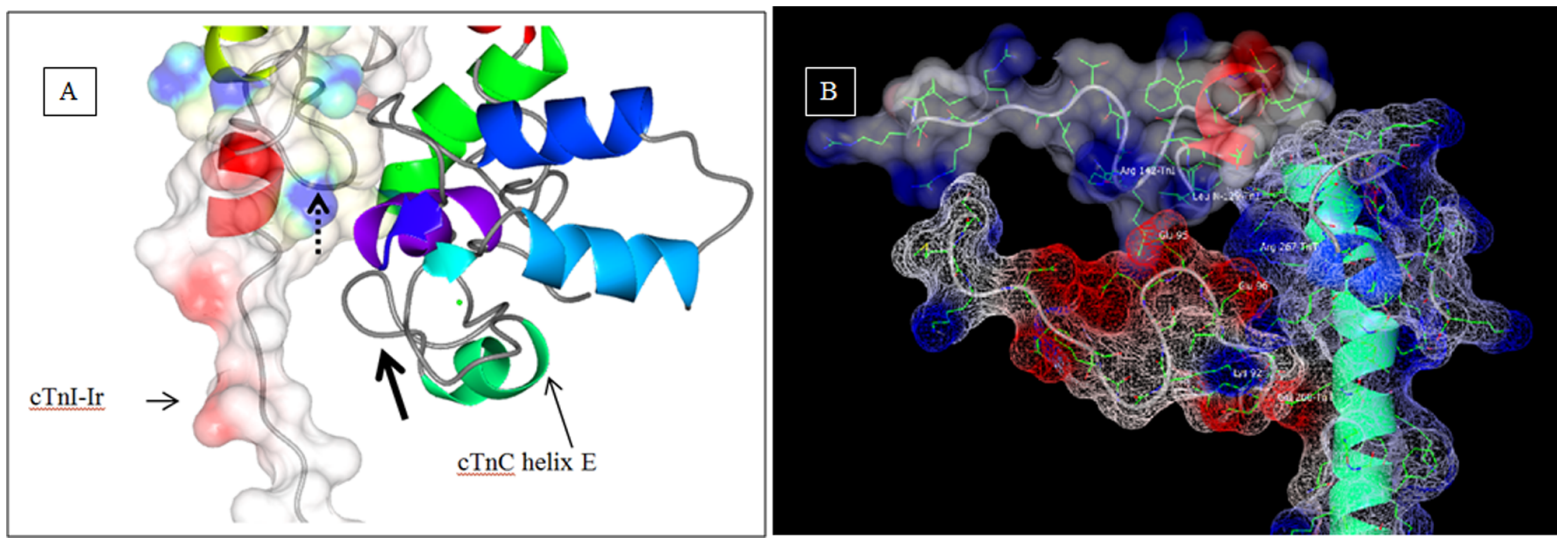
Electrostatic surface analysis of the cTnC and cTnI. Depicts the electrostatics in the vicinity of cTnC helix E in the Ca^2+^-free state after 9.5 ns of simulation. The hydrophobic, negative and positive surfaces are colored white, red and blue respectively. (a) The thick arrow points to the unfolded segment in helix E. The positively charged blue residues of cTnI-Ir are seen arching (pointed to by the dotted line with arrow head) towards the unfolded cTnC helix E (pointed to by thick black arrow). The amino acids sequence of the cTnI-Ir residues is 138-KFKRLPT and the sequence of the opposing cTnC residues are 92-KSEEEL. The predominantly negative (red) cTnC helix E is attracted to the positive region of cTnI-Ir. The unfolded helix E has adopted a “U” shape (pointed to by the thick black arrow). (b) The unfolded helix E is seen in concert with cTnI and cTnT. The cTnC Glu94 is attracted to cTnI Lys141 (not seen in picture), cTnC Glu95 is attracted to the nitrogen on Leu129 of cTnI and Arg142 of cTnI, and cTnC Glu96 is attracted to Arg 267 of cTnT.

As we traverse towards the D/E linker region, in the Ca^2+^-free state the D/E linker residues N-terminus of Asp87 were positioned towards the cTnI-Ir. We postulate that the folding and unfolding of helix D residues 82–86 in response Ca^2+^ flux pulls and releases the D/E linker residues, which in turn translate to the unfolding and refolding of cTnC helix E. The unfolded helix E residues (94–97) in the Ca^2+^-free state, were seen arching towards the cTnI-Ir (138–144) (Figure 6). We postulate that the unfolding of helix E probably helps control the release of the cTnI inhibitory region to interact with actin in the thin filament. Closer examination of the electrostatic surface of the troponin complex revealed that the unfolded region of cTnC is predominantly negative with three Glu residues. These negative residues were attracted to positively charged residues on TnI (Figure 6). It is known that the flexibility of the central helices of cTnC have a significant functional role in muscle regulation as cTnC mutants with altered central linker lengths or reduced linker flexibility are ineffective in the regulation of actomyosin ATPase [30]–[32]. The observed Ca^2+^-induced changes in the structural dynamics of the central helices of cTnC may provide molecular basis of Ca^2+^ signaling in the cardiac thin filament regulation. We postulate that the Ca^2+^ flux based folding and unfolding of helix D of cTnC propagates the Ca^2+^ signal to cTnI-Ir via the D/E linker of cTnC, which adopts a flexible conformation in the Ca^2+^-free state and adopts a rigid conformation in the Ca^2+^-saturated state. The alternating flexibility of the D/E linker regions probably releases and retracts the cTnI-Ir, towards or away from actin.

Another noticeable feature from comparing the two final structures of the distance restrained simulations is that removal of Ca^2+^ from the regulatory binding site of cTnC in the troponin complex not only led to a flexible D/E linker of cTnC, but also caused the N-domain of cTnC to rotate clockwise with respect to the C-domain by 40 degrees. The rotation commences from Asp87 of the central D/E linker and is most conspicuous in the angle of helix D. In the Ca^2+^-saturated state the cTnC helix D is nearly 70 degrees to the normal whereas and in the Ca^2+^-free state it dropped to 30 degrees. The rotated N-domain was stabilized by an intra-protein hydrogen bond network formed between helix N and helices D and E of cTnC (Table 2). The rotation of the N-domain of troponin C was observed in the Ca^2+^-free state of the skeletal troponin complex [25], [26]. The observed Ca^2+^-induced changes in the central linker of cTnC and rotation of the helix D are corroborated by the results of a recent study of cTnC using paramagnetic relaxation enhancement NMR [33]. Their study revealed that in solution cTnC has considerable interdomain flexibility. Also they found that in the Ca^2+^- saturated state there was a rotation of the helix D in the counter clock wise direction, indicating a recovery from a clockwise rotation. Also structural features of the isolated cTnC are retained in our troponin complex modeling studies. In the Ca^2+^ free crystal structure of cardiac troponin C, 1SPY.pdb [29], the helix D is tilted with respect to the structure of helix D found in the Ca^2+^-saturated crystal structure, 1MXL.pdb [7]. Also the direction of the tilt is similar to what is seen in our Ca^2+^-free state simulations.

### Structure of the cardiac troponin I

The cardiac troponin I has a unique N-terminal extension that is not present in its skeletal counterpart and plays an important role in modulating cardiac function. In the crystal structure of the cardiac troponin core domain, no structural information of this region was determined [10]. In our final distance restrained simulation structures, the unique N-terminal extension of cTnI was shown partially over the cTnC N-domain and interacts with sites 1 and 2 of cTnC via hydrogen bonds. These interactions with sites 1 and 2 are not sensitive to Ca^2+^. This location of the N-terminal extension is consistent with previous reports. For example, previous NMR experiments [14] had shown interaction between the N-terminal extension of cTnI and cTnC sites1 and 2. Also the interaction of the N-terminal extension of cTnI with the site1 residues of cTnC was previously detected by peptide array studies [17].

Another important functional region of cTnI in regulating cardiac function is the inhibitory region of cTnI. This was missing in the crystal structure. In our simulations, the cTnI-Ir was rigid and alongside the DE linker in the Ca^2+^-saturated state, whereas in the Ca^2+^-free state it had become floppy and moved upwards in space towards helix C. In the Ca^2+^-free state the cTnI-Ir was positioned away from the D/E linker and interacted with cTnC helix C via hydrogen bonds. Since in this modeling study we do not have actin nor tropomyosin, the cTnI-Ir translocation towards actin is not seen, but the structural displacement, hints that this region is mobile in the Ca^2+^-free state. To further validate the dynamics of the cTnI-Ir revealed in our models, we compared the distances associated with cTnI-Ir in our models to previously determined FRET distances [13], [34]. These FRET distances were not incorporated in this modeling study. The distance changes measured between cTnI residues 129 and 151 and the distance changes measured between the cTnI-Ir to cTnC 89 in the model are comparable to the FRET distances (Table 4).

**Table 4:** Comparison of measured FRET distances against model and crystal structure.

cTnI	cTnI	FRET distance [54]		MD model		crystal structure (PDB ID:1J1E)	
		Mg (Å)	Ca (Å)	Mg (Å) 9.5 ns	Ca (Å) 11.1 ns	Mg (Å)	Ca (Å)
L129	S151	26.8(5.0)	32.5(4.6)	24.64	30.08	—	38.87

cTnC	cTnI	FRET distance [13]		MD model		crystal structure (PDB ID:1J1E)	
		Mg (Å)	Ca (Å)	Mg (Å) 9.5 ns	Ca (Å) 11.1 ns	Mg (Å)	Ca (Å)
S89	S151	26.4(12.4)	19.9(7.1)	24.86	19.39	—	16.26

In the table above the distances from our model are compared against previously measured FRET distances [13], [54]. Also the distances in the Ca^2+^-saturated state crystal structure are provided for comparison [10]. The Mg refers to the Ca^2+^-free state and Ca refers to the Ca^2+^-saturated state. The FRET distances in the table were not incorporated in the simulations and hence serve as a check to see if the model generated is valid. In the FRET analysis, due to the ambiguity in the value of the dipole–dipole orientation factor between energy donor molecules and energy acceptor molecules and due to the dimensions of the probes attached by linkers to the side chains of the amino acid residues, the measured distance will have an uncertainty of ±10% [53]. Although the length of probe linkers is ∼10 Å, the linker is not unidirectional but folds randomly (during folding and rotation the probe and can acquire length of ∼7 Å).

Based on these above factors there is good correlation between the measured FRET distances and the model. The value within the bracket pertains to the half-widths.

**Figure 7:**
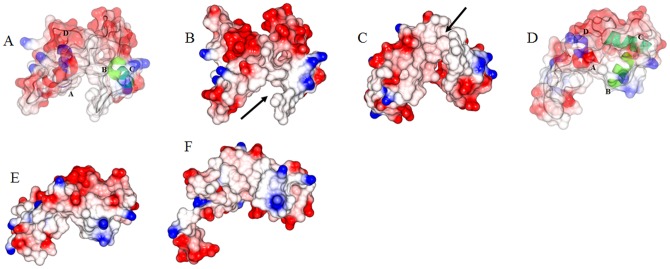
Electrostatic surface analysis of the N-domain of cTnC. Depicts the N-domain of cTnC in the Ca^2+^-free state (figures a through c) and Ca^2+^-saturated states (figures d through f). The cTnC N-domain helices A through D are colored as red, green, cyan and blue respectively. (a) Transparent rendering of the electrostatic surface is shown with the protein in cartoon. (b) In the Ca^2+^-free state the loss of regulatory Ca^2+^ caused the rearrangement of helices B and C. These helices are no longer orthogonal to each other but nearly parallel. This resulted in a breach in the hydrophobic pocket that surrounded the cTnI-Rr (pointed out by the arrow). (c) The cTnC N-domain hydrophobic pocket when viewed from below the cTnC N-domain. This view shows the hydrophobic environment in which the cTnI-Rr is located. The arrows points to the gap in the hydrophobic pocket. (d) Transparent rendering of the electrostatic surface with the protein rendered as cartoon. (e) In the Ca^2+^-saturated state there is no breach in the hydrophobic pocket within which the cTnI-Rr is held. (f) The cTnC N-domain hydrophobic pocket in the Ca^2+^-saturated state when view from below.

**Figure 8:**
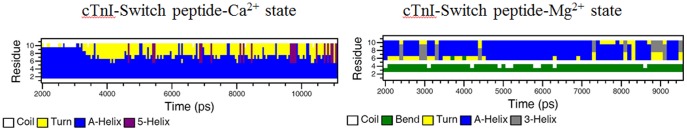
Secondary structure timeline of the cTnI-Rr/switch. The cTnI-Rr is held within the cTnC N-domain hydrophobic pocket. In the presence of regulatory Ca^2+^ the cTnI-Rr maintains a helical conformation. In the absence of regulatory Ca^2+^ the secondary structure is perturbed. The absence of the helical conformation would release the cTnI-Md to interact with actin in the Ca^2+^-free state whereas, the presence of the regulatory Ca^2+^ would refold the cTnI-Rr into a helix effectively retracting the cTnI-Md from actin.

Another interesting observation in our modeling study pertains to the cTnI-Rr held within the cTnC N-domain hydrophobic pocket. The cTnI-Rr was dilated in the Ca^2+^-free state but was still held within the cTnC N-domain hydrophobic pocket. It did not drop off the cTnC N-domain hydrophobic pocket as seen in the crystal structure of the sTn Ca^2+^-free state (1YVO.pdb) nor had it moved away from cTnC as suggested by the authors who solved the structure of cardiac troponin core domain [10]. Instead in the Ca^2+^-free state the cTnI-Rr was dilated and had rotated clockwise with the whole N-domain to 80 degrees to the normal (was positioned at 1 and 7 O'clock) and in the Ca^2+^-saturated state it retained it secondary structure and was oriented at 120 degrees to the normal (between 11 and 5 O'clock). It seemed like the cTnI-Rr undergoes a swivel like motion. To understand what could cause the dilation we ran an electrostatic surface analysis of the cTnC N-domain and observed that the reorientation of helix B caused a gap in the hydrophobic pocket within which the cTnI-Rr is held (Figures 7a through 7c). Whereas, in the Ca^2+^-saturated state the cTnC N-domain hydrophobic pocket was intact and the cTnI-Rr held within the hydrophobic pocket was well structured. The secondary structure timeline of the cTnI-Rr is presented in Figure 8. It is clearly seen that the cTnI-Rr does undergo loss of secondary structure in the Ca^2+^-free state with respect to the Ca^2+^-saturated state.

### Ca^2+^ regulation within the cardiac troponin complex

To further understand how the Ca^2+^ signal is transferred from cTnC to cTnI in the troponin complex, we superimposed the two final distance restrained simulated structures of the troponin complex, one is Ca^2+^-free state (Figure 4c) and the other is Ca^2+^-saturated state (Figure 5c). The superimposed structures were orientation such that the IT arm and the C-domain of cTnC are positioned close to the eye and looking along the D/E linker to the cTnC N-domain. By reviewing these superimposed images, in addition to the structural features of cTnC and cTnI that were discussed previously, we observed more changes in troponin structure in response to Ca^2+^. For example, in the Ca^2+^-saturated state the mobile domain of cTnI was positioned away from the cTn complex whereas in the Ca^2+^-free state it had dropped down by ∼45 degrees from the horizontal and relocated toward helix A of cTnC. This large movement of the cTnI-Md does agree with the proposed movement of the C-terminal region of cTnI by the authors who solved the cardiac troponin crystal structure [10].

The cTnT C-terminal residues Asp271-Lys288 experienced unfolding in both the Ca^2+^-free and Ca^2+^-saturated states, it was more unfolded when Ca^2+^ was present. The observed dynamic nature of this region may have implications in the context of regulation in thin filament. If one were to look at the high resolution image of the thin filament previously reported [35]. It is very clearly seen that the tropomyosin is closer to the troponin complex in the Ca^2+^-free state and farther from the troponin complex in the Ca^2+^-saturated state. As per a previous study it was found that the C-terminus of cTnT interacts with tropomyosin [36]. So it would make sense that for the C-terminal of cTnT to interact with tropomyosin, it would have to be more unfolded and positioned away from the cTn complex in the Ca^2+^-saturated state (as seen in our simulated structure) because tropomyosin is farther away in the thin filament scenario, and the C-terminal of cTnT would be less unfolded in the Ca^2+^-free state and positioned closer to the cTn complex (also seen in our simulations) because tropomyosin would be closer to the cTnT in the Ca^2+^-free state.

Furthermore, a Ca^2+^-induced anharmonic motion of the residues 202–223 at the N-terminus of cTnT was observed in the superimposed images. When Ca^2+^ was absent, the structure had moved away from the Ca^2+^-saturated state by ∼70 degrees (seen at the bottom of the IT arm in Figures 4a and 5b). This anharmonic movement would be meaningful only in the context of the thin filament. It might be misleading to postulate on their dynamics of this segment, with respect to only the cTn complex, because cTnT extends away from the cTn complex by another 201 amino acids. The only comment that we can make is based on the electron microscopy reconstruction of the TF [35] (we thank Prof. Lehman for graciously sharing the reconstruction) where a density is observed beneath the cTn complex in the Ca^2+^-saturated state but is absent in the Ca^2+^-free state. This segment might be experiencing Ca^2+^ based fluctuations in the TF, which needs to be further investigated.

With all these pieces of information, we propose a sequence of events that would lead to cardiac muscle relaxation from the activated state. The loss of regulatory Ca^2+^ is the first in the sequence of events that leads to cardiac muscle relaxation. The loss of regulatory Ca^2+^ opens up a two pronged pathway by which the Ca^2+^ signal is transmitted from cTnC to, the cTnI-Md and the cTnI-Ir. Dissociation of Ca^2+^ from the regulatory binding site of cTnC shifts the dynamic equilibrium of the N-domain of cTnC to the closed state [27], which is accompanied by conformational changes of helices B and C and movement of helix B closer to helix A. The closing of the cTnC N-domain hydrophobic pocket, coupled with the dilation and change in orientation of the cTnI-Rr held within the hydrophobic pocket would deploy the cTnI-Md to bind to actin. Concomitantly the rotation of the cTnC N-domain combined with the folding of helix D would destabilize the D/E linker and the release the cTnI-Ir to move towards actin. Upon analyzing the RMSF plots (Figures 3c, S3, and S6) it is clearly seen that the cTnI-Ir does not exhibit the large fluctuation as the cTnI-Md does. This leads us to conclude that cTnI-Md is the region that aggressively associates with actin rather than cTnI-Ir on a beat to beat basis. Whereas cTnI-Ir might tend to go towards actin but it may not specifically bind to actin. The structural dynamics of cTnI-Ir might be better visualized when the thin filament is modelled. The binding of cTnI-Md to actin would push tropomyosin towards the blocked state on the actin surface. The interaction of the C-terminal residues of cTnT to tropomyosin would lock the tropomyosin in the blocked state. Upon binding of regulatory Ca^2+^ the above said features are reversed. The binding of regulatory Ca^2+^ would reverse the clockwise rotation of the cTnC N-domain and cause conformational changes of helices B (wherein helix B being parallel to helix C in the Ca^2+^-free state is now orthogonal to helix C in the Ca^2+^-saturated state) and open the cTnC N-domain hydrophobic pocket. This would cause the cTnI-Rr held within the cTnC N-domain hydrophobic pocket to regain its secondary structure and undergo a change in orientation. This would retract the cTnI-Md from actin. Concomitantly the refolding of helix E would make rigid the D/E linker region and retract the cTnI-Ir towards the troponin complex, thereby initiating cardiac muscle contraction.

To summarize, the Ca^2+^ fluctuations cause the cardiac troponin complex to take two separate pathways which result in either muscle contraction or muscle relaxation. We used FRET distances and half-widths to model the cardiac troponin complex in the Ca^2+^-free and Ca^2+^-saturated states. This approach helped us to compare the structures in either biochemical states and come up with a model of how the Ca^2+^ signal received at cTnC is transferred to cTnI.

## Materials and Methods

### Protein preparation

The wild-type recombinant cTnC was over-expressed in E. coli strain BL21 (DE3) cells and purified as previously described [6], [18]. A mouse cDNA clone of cTnI was used to generate single-cysteine mutants. Since the wild type has two endogenous cysteines at positions 81 and 98, these residues were converted to Ser and Ile, respectively. Similar mutations were previously found to have a minimal effect on the functional properties of cTnI [22]. Triple mutations were required to generate a single-cysteine rat cardiac TnI mutant at the following positions: S5C, A15C, A17C, Y27C, Y30C, S40C, S43C, Q131C, L145C, A151C, L160C and S167C. The mutant clone construction, protein expression, purification, and characterization were previously described [22]. An adult rat cTnT clone was sub cloned into a pET-17b vector and expressed in BL21 (DE3) cells as previously described [37], [38]. Three single-cysteine cTnT mutants were generated: S240C, S276C, and W288C. Four rat cardiac cTnC clones with single cysteine mutations at 12, 35, 89 and 93 were used to generate cTnC mutants. They were purified and characterized as previously reported [39] The tropomyosin (Tm) was purified from bovine cardiac tissue as previously described [40].

Two pairs of sulfhydryl donor-acceptor probes were used for FRET measurements between cTnI and cTnT: (a) AEDANS-DABMI (4-Dimethylaminophenylazophenyl-4′-Maleimide) and (b) MIANS-DDPM. The second pair of probes was used to measure two distances, TnI 131 to TnT 276 and TnI 131 to TnT 278, and the first pair was used to measure all other distances. For distance measurements between cTnC (residues 12, 35, 89, 93) and cTnI (residues 5, 15, 30, 43) the AEDANS-DDPM pair was used. The procedure for sulfhydryl labeling of the proteins have been previously described [22]. The degrees of labeling with the probes were >0.97 mol probe/mol of protein. A mouse cDNA clone of cTnI was used to generate a mutant cTnI containing a single tryptophan residue at the position 129 or 150 and a single cysteine residue at the position 5, or 15, or 30, or 43. In the mutant, the endogenous Trp192 was changed to Phe and the two endogenous Cys81 and Cys98 were mutated to Ser and Ile, respectively. Similar mutations were previously found to have a minimal effect on the functional properties of cTnI [37]. The mutant clone construction, protein expression, purification, and characterization were carried out as in our previous work [37]. The single cysteine residue in the cTnI mutant was labeled with IAEDANS in the presence of 6 M urea [22] and degree of labeling was found to be >0.97 mol probe/mol of protein..

Reconstitution of cTn with cTnC, donor-labeled cTnI, and acceptor-labeled cTnT was carried out in 6 M urea at a molar ratio of TnC∶TnI∶TnT = 1.5∶1.0∶1.2, following a previous procedure [18]. To examine the stability and functional effects of cTn protein mutations and probe modifications, SDS-PAGE and native gel analysis, and Ca^2+^ regulation of the actin-activated S1-ATPase activity assay was carried out as described previously [41]–[43]. The results (data not shown) show no evidence of protein degradation and suggest that the functional effects of the mutations and modifications of proteins on the Ca^2+^ regulatory activity were negligible.

### Fluorescence measurements

The steady-state fluorescence measurements were made at 20°C on an ISS PC1 photon-counting spectrofluorometer using a band pass of 3 nm on both the excitation and emission monochromators with samples (2 µM protein) in the working buffer (30 mM Mops (3-(N-mopholino)propanesulfonic acid) at pH 7.0, 5 mM MgCl_2_, 0.15M KCl, 1 mM DTT, and 1 mM EGTA(ethylene glycol-bis-(β-aminoethyl ether)-N,N,N′,N′-tetraacetic acid)) in the presence and absence of 3 mM CaCl_2_. The emission spectra were corrected for variation of the detector system with wavelength. The donor quantum yields were determined by the comparative method using quinine sulfate as the standard [39]. The measured quantum yields were used in the calculation of R_0_, the Förster critical distance at which the efficiency of energy transfer is 0.5. In this calculation, a value of ⅔ was used for the orientation factor κ^2^.

Time-resolved fluorescence measurements were carried out at 20°C with samples (2 µM protein) in the working buffer or in the buffer containing 3 mM CaCl_2_. The fluorescence intensity decays were measured in the time domain with a time-correlated single photon-counting lifetime spectrometer. The samples containing AEDANS as donor were excited with 348 nm LED light and the emission was isolated with a 410 nm cut-off filter, while the samples containing tryptophan as donor were excited with 295 nm LED light the emission was isolated with a 340 nm interference filter. The intensity decay of the donor from the donor-alone and the donor-acceptor samples were each collected into 1024 channels of a multichannel analyzer under identical conditions. The decays were analyzed by an exponential model. The distance-dependent donor decay of a donor-acceptor pair separated by a given distance r is given by
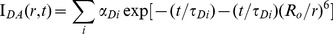
(1)Where τ_Di_ is the decay time of the donor in the absence of acceptor, α_Di_ is the associated amplitude, and R_0_ is the Förster distance. The observed decay for an ensemble of donor-acceptor pairs is given by
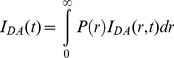
(2)where P(r) is the probability distribution of the distances and is assumed to be a Gaussian function with the mean distance r and half-width (HW) = 2.35σ, where σ is the standard deviation of the distribution. If the donor-acceptor pair was rigidly held in space, the distribution would be a spike with a very small half width. The distribution was calculated from Equation (2) using the program CFS_LS/GAUDID [22]. For each donor-acceptor pair and each pair of mutants, R_0_ was experimentally determined for each set of experimental conditions. The reduced chi-squares ratio (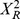) and the residual plot were used to judge the goodness of fit for the calculated distribution of distances.

### Docking and molecular dynamics simulations

The coordinates of the cTn complex (PDB ID-1J1E) [10] and the N-terminal extension of cTnI (PDB ID-2JPW) [16] were retrieved from the protein data bank. The crystallographically unresolved loop regions in the cTn complex (the cTnI-Ir and residues connecting helices B and C of cTnC) were built using the modloop server [44], [45]. The missing residues 276–288 on the C-terminus of cTnT were built as a helix using Whatif [46]. The rationale behind building this region as a helix was that with the force field and distance restraints in place it would unfold and assume a conformation which is biologically relevant. Also modeling the C-terminal of cTnT as a helix eliminated spurious interaction between helix C of cTnC with the N-terminal extension of cTnI. Hex (version 6.3), protein-protein rigid body docking software, was used to dock the 32 mer N-terminal extension of cTnI (ligand) on the surface of the cTn complex (receptor) [47]. Full rotation, shape and electrostatic docking of the ligand to the receptor were carried out. The grid dimensions, receptor range, ligand range and twist range values were set to default values and the post-docked structures were subjected to molecular mechanics minimization. The docked structures were visually examined and only those structures where the N-terminal extension of cTnI was positioned above the cTnC N-domain were retained. The phosphorous atoms on the N-terminal extension of cTnI in the original coordinate file [16] were deleted and the ligand was ligated to the cTn complex using modloop server.

In preparation for MD simulations the protein was placed in a triclinic box filled with 71739 SPC water molecules and set to physiological pH. The salt concentration was set at 0.15M NaCl to mimic experimental salt concentrations and sodium counter ions were added to maintain electroneutrality. The MD simulations were carried out on Cheaha cluster at the UAB IT Research Computing, University of Alabama at Birmingham, Alabama, USA. The GROMOS96 43a1 force field embedded in GROMACS [48] was used for all simulations. Steepest descents and leap-frog algorithms were used during EM and MD simulations. The FRET distances and half widths from Tables 1 were applied as distance restraints and bond energies between the C_α_'s of the participating residues during EM, SA and MD simulations as previously described [49]. The lower and upper limits for the distance restraints were set with the measured FRET distances and FRET distance plus 7 Å, respectively. In our simulation the distance restrains were set between the C-alphas of proteins whereas FRET distances are measured between benzene rings of probes that were attached to proteins through a 7 Å linker. To apply the FRET distances as restrains in the simulation we need not only to consider contributions of the linker length, but also the “swooping” cone orientations of two probes. Depending on the surface environments of the labeling sites, the two cones could orientate toward each other (which will underestimate the real distance) or orient opposite each other (which will overestimate the distance), or they may be oriented in different directions. The orientations of these probes could not be experimentally determined. Therefore, in an effort to account for these contributions we used FRET-7 and FRET +7, as well as just FRET distance, as the lower and upper limits of the distance restraints during our initial modeling trials. The rationale of these modeling trials was that the obtained model under each FRET constrain must replicate experimentally determined features. For example, the opening or closing of the cTnC N-domain hydrophobic pocket would take place in response to the presence or absence of regulatory Ca^2+^ in site 2 of cTnC. In our simulations structure displayed in figure 2 exhibited this feature. Results of these modeling suggest the model of the cardiac troponin complex obtained by having the FRET distance set as the lower limit and FRET+7 Å as the upper limit were biologically relevant.

During the restrained MD simulations, during the initial 200 ps, SA was performed on the N-terminal extension of cTnI. During the SA the temperature of the whole system was maintained at 300 K with the exception of the 40 amino acids that comprises the N-terminal extension of cTnI (1–40 amino acids). The temperature of the N-terminal extension of cTnI was increased in increments of 50 K from 300 K to 500 K and lowered to 300 K over a period of 200 ps. During the simulations the neighborhood list was updated every 10 steps and the bonds were not constrained during EM, SA and MD simulations. The electrostatic interactions were computed using PME (Particle Mesh Ewald). The nonbonded interactions were treated with a cut-off commencing at 10 Å and reaching zero at 14 Å. The simulations were performed under constant temperature and pressure (P = 1 atm; T = 300 K) [50]. The time step for integration was 0.001 ps for the distance restrained simulations. During the free simulations all the conditions in which the restrained MD simulations were maintained, except that there were no distance restraints applied nor was any SA performed, the time step for integration was 0.002 ps and all the bonds were restrained by LINCS

The structures were viewed using Rasmol [24] and the graphics were prepared using Pymol (www.pymol.org) and CCP4MG package [51], [52].

## Supporting Information

Figure S1 **The opening and closing of the cTnC N-domain hydrophobic pocket in simulations II.** The distance between the cTnC residues 13 and 51 are plotted as a function of time. The Ca^2+^ saturated and Ca^2+^-free states are colored red and blue respectively.
(TIF)

Figure S2 **The distance between cTnC residues 13 and 51C monitored over 250 ns of simulations.** Monitoring the distance between the two cTnC residues 13 and 51 helped reveal the cTnC N-domain hydrophobic pocket fluctuations between open and closed states. After allowing the initial 25 ns for equilibration, based on the minimum distance between the cTnC residues 13 and 51, we can say the time the Ca^2+^ saturated state structure spent in the closed state was from 67–70 ns, 81–111 ns and from 124–136 ns which is ∼18% of the simulation time. Likewise the time the Ca^2+^-free state spent in the open state was from 79–109 ns, 114–122 ns, 127–137 ns, which is ∼19.2% of the simulation time.
(TIF)

Figure S3 **RMSF of the cTn complex simulated for 250 n after the distance restraints were released (simulations II).** The root mean square fluctuation of the cardiac troponin complex was calculated over 250 ns. In the graph the C-alphas from 1–161 pertain to cTnC, 162–249 pertain to cTnT, 250–442 pertain to cTnI. In the Ca^2+^-saturated state fluctuations of more than 4 Å are observed from C-alpha 235–249. These pertain to the residues in the C-terminal end of cTnT helix H2 (residues 274–288). In the same state, the C-alphas 264–280 (correspond to residues 14–30 of cTnI) correspond to the N-terminal extension of cTnI. Towards the end of the x-axis we can see that the C-terminal end of cTnI (pertains to the cTnI-Md) experiences fluctuations in both the biochemical states. In the Ca^2+^-saturated state the C-alphas from 412–442 (corresponding to cTnI-Md residues 161–191) experience fluctuation, whereas in the Mg^2+^ (Ca^2+^ free) state the C-alphas from 425–442 experience fluctuation (correspond to cTnI-Md residues 174–191).
(TIF)

Figure S4 **The opening and closing of the cTnC N-domain hydrophobic pocket (simulations III).** The cTn complex was simulated in the absence of any distance restraints. The distance between the cTnC residues 13 and 51 was plotted as a function of time. The Ca^2+^-saturated and Ca^2+^-free systems are colored burgundy and sky blue.
(TIF)

Figure S5 **RMSD of the protein in simulation III.** The cTn complex was simulated without distance restraints. The RMSD of the cTn complex was plotted as a function of time. The Ca^2+^-saturated and Ca^2+^-free systems are colored burgundy and sky blue.
(TIF)

Figure S6 **RMSF of the protein in simulation III.** The root mean square fluctuations of the cardiac troponin complex were calculated for the cTn complex which was simulated for 150 ns without any restraints. In the graph the C-alphas from 1–161 pertain to cTnC, 162–249 pertain to cTnT, 250–442 pertain to cTnI. Fluctuations of more than 3 Å are observed in the N-terminal helix H1 of cTnT in both the Mg^2+^ (Ca^2+^-free) and Ca^2+^-saturated states. The C-alphas 162–177 in the graph pertain to cTnT residues 202–217 in the crystal structure). Fluctuations are observed at the N-terminal extension of cTnI (C-alphas 251–280, they pertain to cTnI residues 1–30). Towards the end of the x-axis fluctuations are observed in the C-terminal end of cTnI (C-alphas 415–442). These pertain to residues 164–191 of the cTnI-Md that are experiencing fluctuations in both the biochemical states.
(TIF)

Figure S7 **Structure of the cTn complex in the Ca^2+^-free and Ca^2+^-saturated states after 150 ns of simulations.** (a) Depicts the structure of the cTn complex in the Ca^2+^-free state after 150 ns of simulations. (b) The structure of the cTn complex in the Ca^2+^ saturated state after 150 ns of simulations. The cTnC N-domain helices have not unfolded because no distance restraints were in place.
(TIF)

File S1 (DOCX)
