# Supplementary figures and images for: Molecular Dynamics Simulations of the Cardiac Troponin Complex Performed with FRET Distances as Restraints
Source: PLoS One. 2014 Feb 18;9(2):e87135. doi: 10.1371/journal.pone.0087135 (PMC3928104; doi:10.1371/journal.pone.0087135)

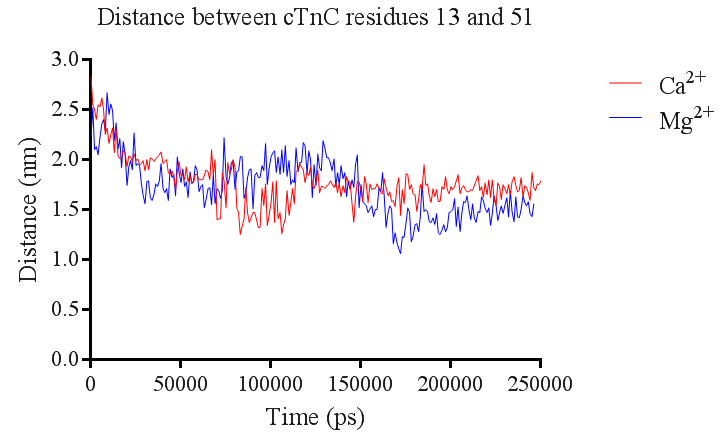

Supplement: Figure S1 — The opening and closing of the cTnC N-domain hydrophobic pocket in simulations II. The distance between the cTnC residues 13 and 51 are plotted as a function of time. The Ca2+ saturated and Ca2+-free states are colored red and blue respectively. (TIF) [file pone.0087135.s001.tif]

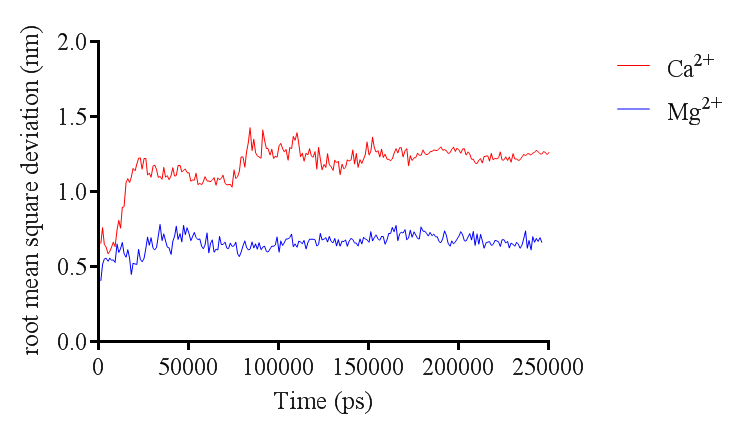

Supplement: Figure S2 — The distance between cTnC residues 13 and 51C monitored over 250 ns of simulations. Monitoring the distance between the two cTnC residues 13 and 51 helped reveal the cTnC N-domain hydrophobic pocket fluctuations between open and closed states. After allowing the initial 25 ns for equilibration, based on the minimum distance between the cTnC residues 13 and 51, we can say the time the Ca2+ saturated state structure spent in the closed state was from 67–70 ns, 81–111 ns and from 124–136 ns which is ∼18% of the simulation time. Likewise the time the Ca2+-free state spent in the open state was from 79–109 ns, 114–122 ns, 127–137 ns, which is ∼19.2% of the simulation time. (TIF) [file pone.0087135.s002.tif]

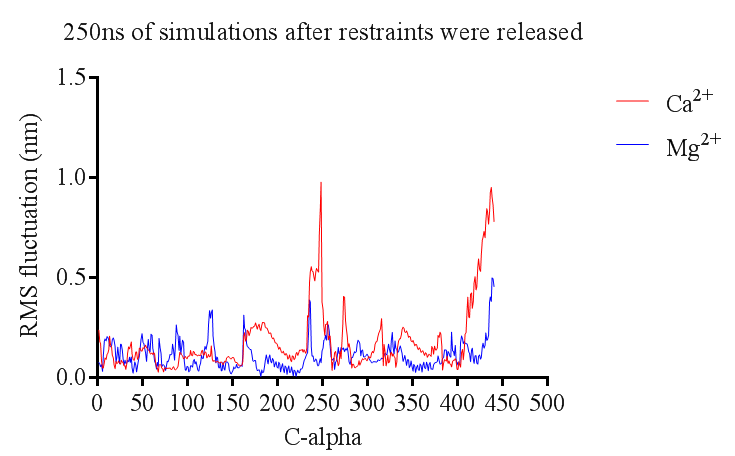

Supplement: Figure S3 — RMSF of the cTn complex simulated for 250 n after the distance restraints were released (simulations II). The root mean square fluctuation of the cardiac troponin complex was calculated over 250 ns. In the graph the C-alphas from 1–161 pertain to cTnC, 162–249 pertain to cTnT, 250–442 pertain to cTnI. In the Ca2+-saturated state fluctuations of more than 4 Å are observed from C-alpha 235–249. These pertain to the residues in the C-terminal end of cTnT helix H2 (residues 274–288). In the same state, the C-alphas 264–280 (correspond to residues 14–30 of cTnI) correspond to the N-terminal extension of cTnI. Towards the end of the x-axis we can see that the C-terminal end of cTnI (pertains to the cTnI-Md) experiences fluctuations in both the biochemical states. In the Ca2+-saturated state the C-alphas from 412–442 (corresponding to cTnI-Md residues 161–191) experience fluctuation, whereas in the Mg2+ (Ca2+ free) state the C-alphas from 425–442 experience fluctuation (correspond to cTnI-Md residues 174–191). (TIF) [file pone.0087135.s003.tif]

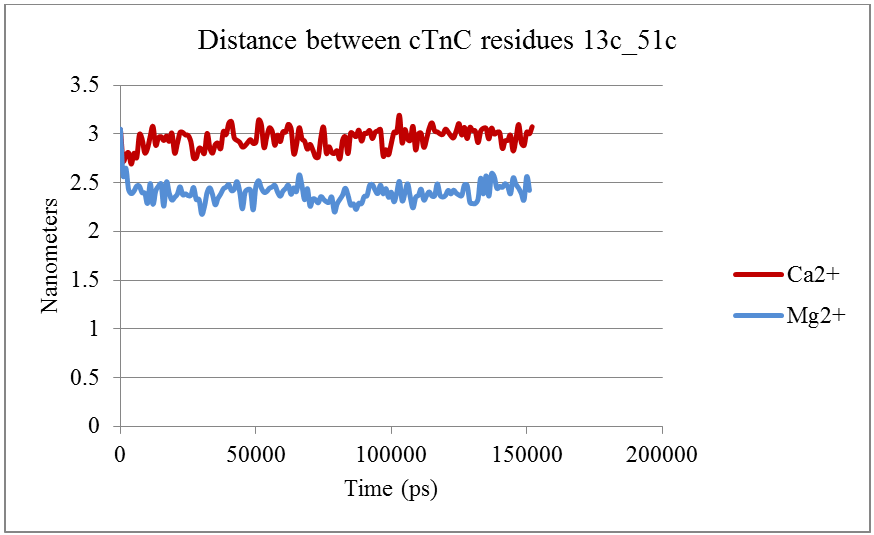

Supplement: Figure S4 — The opening and closing of the cTnC N-domain hydrophobic pocket (simulations III). The cTn complex was simulated in the absence of any distance restraints. The distance between the cTnC residues 13 and 51 was plotted as a function of time. The Ca2+-saturated and Ca2+-free systems are colored burgundy and sky blue. (TIF) [file pone.0087135.s004.tif]

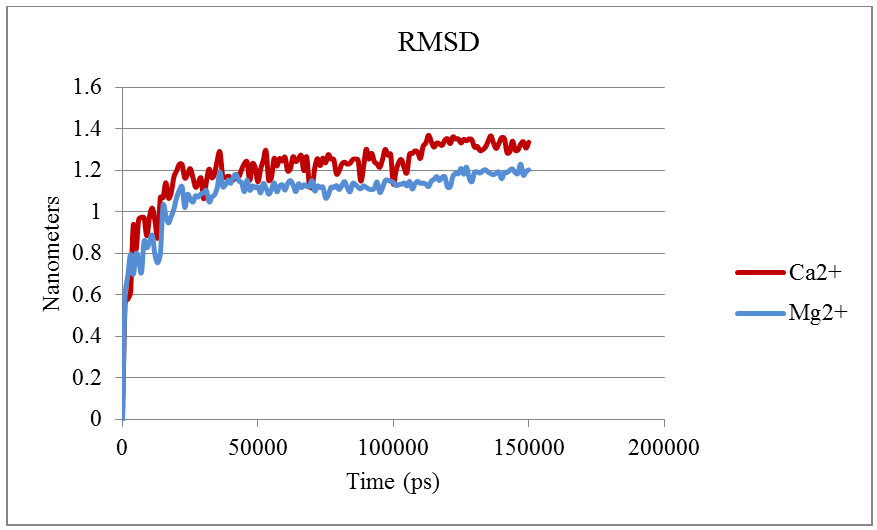

Supplement: Figure S5 — RMSD of the protein in simulation III. The cTn complex was simulated without distance restraints. The RMSD of the cTn complex was plotted as a function of time. The Ca2+-saturated and Ca2+-free systems are colored burgundy and sky blue. (TIF) [file pone.0087135.s005.tif]

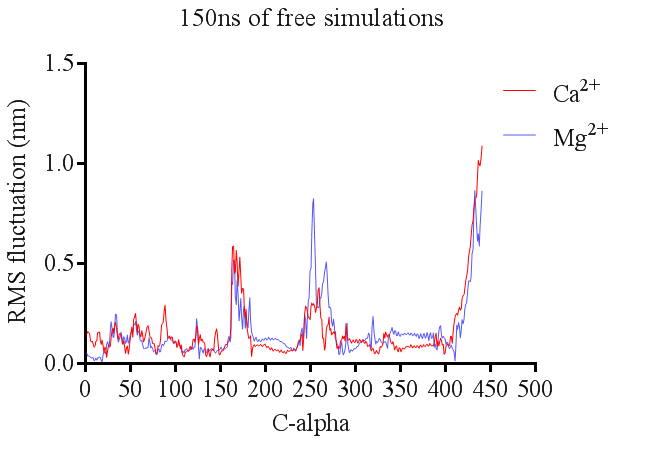

Supplement: Figure S6 — RMSF of the protein in simulation III. The root mean square fluctuations of the cardiac troponin complex were calculated for the cTn complex which was simulated for 150 ns without any restraints. In the graph the C-alphas from 1–161 pertain to cTnC, 162–249 pertain to cTnT, 250–442 pertain to cTnI. Fluctuations of more than 3 Å are observed in the N-terminal helix H1 of cTnT in both the Mg2+ (Ca2+-free) and Ca2+-saturated states. The C-alphas 162–177 in the graph pertain to cTnT residues 202–217 in the crystal structure). Fluctuations are observed at the N-terminal extension of cTnI (C-alphas 251–280, they pertain to cTnI residues 1–30). Towards the end of the x-axis fluctuations are observed in the C-terminal end of cTnI (C-alphas 415–442). These pertain to residues 164–191 of the cTnI-Md that are experiencing fluctuations in both the biochemical states. (TIF) [file pone.0087135.s006.tif]

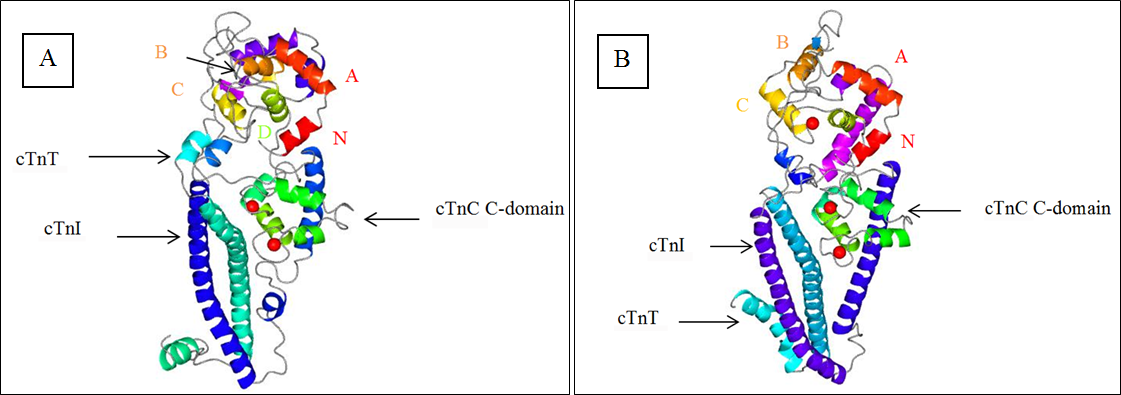

Supplement: Figure S7 — Structure of the cTn complex in the Ca2+-free and Ca2+-saturated states after 150 ns of simulations. (a) Depicts the structure of the cTn complex in the Ca2+-free state after 150 ns of simulations. (b) The structure of the cTn complex in the Ca2+ saturated state after 150 ns of simulations. The cTnC N-domain helices have not unfolded because no distance restraints were in place. (TIF) [file pone.0087135.s007.tif]
