# Supplementary material for: Molecular Dynamics Simulations of the Cardiac Troponin Complex Performed with FRET Distances as Restraints
Source: PLoS One. 2014 Feb 18;9(2):e87135. doi: 10.1371/journal.pone.0087135 (PMC3928104; doi:10.1371/journal.pone.0087135)
Supplement: File S1 — (DOCX) [file pone.0087135.s008.docx]

**SUPPORTING DATA**

Apart from the distance restrained simulations the cTn complex there were two other simulations that were performed.

(1) The terminal structures obtained in the Ca^2+^ saturated (11.1ns) and Ca^2+^-free states (9.5ns), at the end of the distance restrained simulations were subjected to unrestrained simulations for 250ns. This is notated as simulations II.

(2) The starting structure seen in figure 2 was simulated in the Ca^2+^-saturated and Ca^2+^-free states, in the absence of any distance restraints for 150ns. This is notated as simulations III.

**Simulations II**

The terminal structures at the end of the distance restrained simulation were further simulated for 250ns without any restraints. The purpose of this simulation was to understand the dynamics of the cTn complex in both Ca^2+^-saturated and Ca^2+^-free states. The distances between the cTnC residues 13 and 51 was used to monitor the cTnC N-domain opening and closing in either biochemical states and is plotted in Figure S1. The simulations showed that during the initial 6ns of Ca^2+^-saturated state simulations the separation distance between cTnC residues 13 and 51 was greater than the Ca^2+^-free state simulations. Later this separation distance slowly decreases. Around 30ns the distance between cTnC 13 and 51 in Ca^2+^-saturated state simulations is greater than the separation distance between the same amino acids in the Ca^2+^ free state simulations. Later around 60ns the Ca^2+^-saturated state shows a distance increase between cTnC residues 13 and 51 with respect to the Ca^2+^-free state simulation. From 70ns to 113ns the distance between the cTnC residues 13 and 51 decrease in the Ca^2+^ state with respect to the Ca^2+^-free state.

The dynamics of the system is such that the despite the presence of bound regulatory Ca^2+^ the cTnC hydrophobic pocket starts to close. The converse occurs in the Ca^2+^-free state. In the Ca^2+^-free state the cTnC N-domain hydrophobic pocket starts to open up despite the absence of any bound regulatory Ca^2+^. The RMSD and RMSF of the system is plotted as Figures. S2 and S3 respectively. This suggests that there is a population of cTnC molecules in either biochemical states that sample the other state. Such dynamics is also seen in a previous study [[1](#_ENREF_1)].

*Conclusion of simulations II*

The opening and closing of the N-domain hydrophobic pocket shows that despite the presence of bound Ca^2+^ ~18% of the population samples the closed state. Likewise ~19.2% of the population of the cTn complex sample the open state despite the absence of bound regulatory Ca^2+^.

**Simulations III**

The cTn structure seen in Fig. 2 was simulated in *the absence of any distance restraints* for 150ns. The reason for this conducting these simulations was to show that the unfolding experienced by the cTnC N-domain helices was a result of distance restraints that were in place during the distance restrained simulations. These simulations also served to show that the force field was set up correctly and that there is no unfolding of cTnc helices when simulated without any restraints. The distance between the cTnC residues 13 and 51, the RMSD, and RMSF of the system in either biochemical states are seen in Figures. S4, S5, and S6 respectively. The structure of the cTn complex in the Ca^2+^-free and Ca^2+^-saturated states after 150ns of simulations are depicted in Figure. S7.

**REFERENCES**

1. Cordina NM, Liew CK, Gell DA, Fajer PG, Mackay JP, et al. (2013) Effects of calcium binding and the hypertrophic cardiomyopathy A8V mutation on the dynamic equilibrium between closed and open conformations of the regulatory N-domain of isolated cardiac troponin C. Biochemistry 52: 1950-1962.

**Supporting information legends**

Figure. S1.The opening and closing of the cTnC N-domain hydrophobic pocket in simulations II.

The distance between the cTnC residues 13 and 51 are plotted as a function of time. The Ca^2+^ saturated and Ca^2+^-free states are colored red and blue respectively.

Figure. S2. The distance between cTnC residues 13 and 51C monitored over 250ns of simulations.

Monitoring the distance between the two cTnC residues 13 and 51 helped reveal the cTnC N-domain hydrophobic pocket fluctuations between open and closed states. After allowing the initial 25ns for equilibration, based on the minimum distance between the cTnC residues 13 and 51, we can say the time the Ca^2+^ saturated state structure spent in the closed state was from 67-70ns, 81-111ns and from 124-136ns which is ~18% of the simulation time. Likewise the time the Ca^2+^-free state spent in the open state was from 79-109ns, 114-122ns, 127-137ns, which is ~19.2% of the simulation time.

Figure. S3. RMSF of the cTn complex simulated for 250n after the distance restraints were released (simulations II).

The root mean square fluctuation of the cardiac troponin complex was calculated over 250ns. In the graph the C-alphas from 1-161 pertain to cTnC, 162-249 pertain to cTnT, 250-442 pertain to cTnI. In the Ca^2+^-saturated state fluctuations of more than 4Å are observed from C-alpha 235-249. These pertain to the residues in the C-terminal end of cTnT helix H2 (residues 274-288). In the same state, the C-alphas 264-280 (correspond to residues 14-30 of cTnI) correspond to the N-terminal extension of cTnI. Towards the end of the x-axis we can see that the C-terminal end of cTnI (pertains to the cTnI-Md) experiences fluctuations in both the biochemical states. In the Ca^2+^-saturated state the C-alphas from 412-442 (corresponding to cTnI-Md residues 161-191) experience fluctuation, whereas in the Mg^2+^ (Ca^2+^ free) state the C-alphas from 425-442 experience fluctuation (correspond to cTnI-Md residues 174-191).

Figure. S4. The opening and closing of the cTnC N-domain hydrophobic pocket (simulations III).

The cTn complex was simulated in the absence of any distance restraints. The distance between the cTnC residues 13 and 51 was plotted as a function of time. The Ca^2+^-saturated and Ca^2+^-free systems are colored burgundy and sky blue.

Figure. S5. RMSD of the protein in simulation III.

The cTn complex was simulated without distance restraints. The RMSD of the cTn complex was plotted as a function of time. The Ca^2+^-saturated and Ca^2+^-free systems are colored burgundy and sky blue.

Figure. S6. RMSF of the protein in simulation III.

The root mean square fluctuations of the cardiac troponin complex were calculated for the cTn complex which was simulated for 150 ns without any restraints. In the graph the C-alphas from 1-161 pertain to cTnC, 162-249 pertain to cTnT, 250-442 pertain to cTnI. Fluctuations of more than 3Å are observed in the N-terminal helix H1 of cTnT in both the Mg^2+^ (Ca^2+^-free) and Ca^2+^-saturated states. The C-alphas 162-177 in the graph pertain to cTnT residues 202-217 in the crystal structure). Fluctuations are observed at the N-terminal extension of cTnI (C-alphas 251-280, they pertain to cTnI residues 1-30). Towards the end of the x-axis fluctuations are observed in the C-terminal end of cTnI (C-alphas 415-442). These pertain to residues 164-191 of the cTnI-Md that are experiencing fluctuations in both the biochemical states.

Figure. S7. Structure of the cTn complex in the Ca^2+^-free and Ca^2+^-saturated states after 150ns of simulations.

(a) Depicts the structure of the cTn complex in the Ca^2+^-free state after 150ns of simulations. (b) The structure of the cTn complex in the Ca^2+^ saturated state after 150ns of simulations. The cTnC N-domain helices have not unfolded because no distance restraints were in place.
